# Physics of animal health: on the mechano-biology of hoof growth and form

**DOI:** 10.1098/rsif.2019.0214

**Published:** 2019-06-26

**Authors:** Ramzi Al-Agele, Emily Paul, Sophie Taylor, Charlotte Watson, Craig Sturrock, Michael Drakopoulos, Robert C. Atwood, Catrin S. Rutland, Nicola Menzies-Gow, Edd Knowles, Jonathan Elliott, Patricia Harris, Cyril Rauch

**Affiliations:** 1 School of Veterinary Medicine and Science, University of Nottingham, College Road, Sutton Bonington LE12 5RD, UK; 2 CIPB, Hounsfield Building, University of Nottingham, College Road, Sutton Bonington LE12 5RD, UK; 3 BL12, Diamond Light Source Ltd, Diamond House, Harwell Science and Innovation Campus, Didcot, Oxfordshire OX11 0DE, UK; 4 The Royal Veterinary College, Hawkshead Lane, Hatfield, Hertfordshire AL97TA, UK; 5 Equine Studies Group, WALTHAM Centre for Pet Nutrition, Melton Mowbray, Leicester LE14 4RT, UK; 6 Department of Anatomy, College of Veterinary Medicine, University of Diyala, Baqubah, Iraq

**Keywords:** working equids, physics of life, ungulates, hoof shapes, hoof pathologies

## Abstract

Global inequalities in economic access and agriculture productivity imply that a large number of developing countries rely on working equids for transport/agriculture/mining. Therefore, the understanding of hoof conditions/shape variations affecting equids' ability to work is still a persistent concern. To bridge this gap, using a multi-scale interdisciplinary approach, we provide a bio-physical model predicting the shape of equids’ hooves as a function of physical and biological parameters. In particular, we show (i) where the hoof growth stress originates from, (ii) why the hoof growth rate is one order of magnitude higher than the proliferation rate of epithelial cells and (iii) how the soft-to-hard transformation of the epithelium is possible allowing the hoof to fulfil its function as a weight-bearing element. Finally (iv), we demonstrate that the reason for hoof misshaping is linked to the asymmetrical design of equids' feet (shorter quarters/long toe) together with the inability of the biological growth stress to compensate for such an asymmetry. Consequently, the hoof can adopt a dorsal curvature and become ‘dished’ overtime, which is a function of the animal's mass and the hoof growth rate. This approach allows us to discuss the potential occurrence of this multifaceted pathology in equids.

## 1. Background

Equids (horses, mules, donkeys) are ‘ungulates’ or single-digit hoofed mammals. As the function of the hoof is to sustain the animal's weight and to balance external stresses during locomotion, hoof pathologies and associated shape variations have important repercussions on equids' health and have puzzled humankind for centuries, with reference being made in Aristotle's writings around 350 BC [1]. While equids are mostly considered as pets in economically advanced countries, the 110 million working equids worldwide involved in mining, transport and agriculture play an important global socio-economic role in developing countries (https://www.thebrooke.org/). Owning a working equid provides financial benefits to families [2] and plays an important role socially [3]. In this context, the health of working equids remains an important problem as chronic hoof misshaping and related conditions are a serious problem in this genus [4,5] and time off work for recovery has a major impact on an owner's income [2].

As perissodactyls, or odd-toed ungulates, horses have a strong hoof capsule, protecting the internal structures of each single-digit foot. This horny structure encloses the distal part of the second phalanx, the distal phalanx and the navicular bone. The adhesion of the hoof to the distal phalange is warranted by the dermo-lamellar junction that, through its hierarchical design, allows a strong adhesion to take place during an abrupt rise in mechanical stresses, e.g. gallop [6–8] (see electronic supplementary material for a summary of the horse foot anatomy).

Abnormal hoof shapes that can be observed as dorsal curvature anomalies (a.k.a. Aladdin's slipper shape) develop over a long period of time and are commonly perceived as a warning sign underlying a past or present disorder associated with biological factors (e.g. hormonal disturbances) and sometimes, physical factors, including the specific loading of a limb [9–11]. However, to what extent can physical and biological factors be integrated together, or what is the weighting to give to either factor in case of hoof deformity, is unclear. Although the biology of keratinized tissues is being unravelled at the cellular, molecular and genetic levels [12–15]; the full understanding of the multi-scale interactions between the physics and biology in these tissues remains in its infancy.

Biomechanical studies of the equid's hoof capsule were the first to gather essential information regarding the stress/strain relationships and viscoelastic properties of the hoof capsule in relation to its morphology by considering the adult hoof as a static piece of tissue [16–23]. However, the hoof is not static but grows continuously over time and the question as to how the growth of a hoof responds to the physical environment has not received any adequate answers. As a result, a number of questions are still lingering, such as (i) how can the growth rate of the hoof capsule be approximately 0.1 mm day^−1^ [24] when keratinocyte cells proliferate at a typical rate approximately 10 µm day^−1^ (approx. 0.01 mm day^−1^)? (ii) How can a hoof capsule with an elastic modulus approximately 10^8^ Pa [21] emerge from soft keratinocyte tissues where single cells have a typical elastic modulus approximately 10^3^ Pa [25,26]? (iii) What are the biological mechanisms promoting the growth stress to allow the hoof to be a weight-bearing element and how do these mechanisms impact the future shape of the hoof? (iv) To what extent is physics involved and can explain hoof deformities?

These questions underline the notion of growth rate/stress and the transformation that the epithelium has to undergo to generate a solid hoof capsule. As a result, there is a need to investigate the dynamical growth of the hoof using a multi-scale approach from the cells to the entire hoof in live animals.

When studying problems at the interface between physics and biology and in particular any dynamical growths, the challenge is to relate a biological growth originating from soft tissues/cells to a physical stress such that problems can be dealt with physics, in turn informing the biology of the process at play. Using a bottom-up approach, we have therefore concentrated on the ‘biology of the early stage of hoof growth’ to deduce the ‘physics of the early stage of hoof growth’ providing a model for the hoof growth stress. This stress incorporated into a biomechanical model of the hoof treated as a solid will untangle the roles of physics (e.g. weight and hoof geometry) and biology (e.g. proliferation and differentiation of keratinocyte progenitor cells) to demonstrate the weakness of this system.

## 2. Material and methods

### 2.1. Sample collections

Hooves were obtained from horses that were not euthanized for research purposes. Horse hooves were collected from the abattoir 1 h post euthanasia following ethical approval by The School of Veterinary Medicine and Science, University of Nottingham. For three-dimensional imaging and histological sampling, PBS was injected through the medial and lateral palmar digital arteries to remove the blood, and tissues were fixed by replacing PBS with a 4% PFA/PBS (Sigma, UK) fixative solution under manual pressure. When needed, biopsies were taken from the dorsal and quarter parts of the coronary band and placed into a 4% PFA/PBS solution fixative solution prior processing. For primary cell isolation, hooves were aseptically cleaned and progenitor keratinocyte cells obtained as described below. All primary cell cultures were performed at 37°C in 5% CO_2_.

### 2.2. Synchrotron imaging of the papillae

To investigate details of the circulatory system and tissues surrounding and within the papillae, a hoof specimen was imaged on Beamline I12-JEEP at the Diamond Light Source, UK [27] using 0.234 Å (53 keV) X-rays and a custom-built X-ray camera, including an X-ray sensitive scintillator emitting visible light (cadmium tungstate), visible light optics and a PCO.edge camera, with scientific grade 2560 × 2160 pixel sCMOS sensor. 1800 images were collected at 0.1° intervals through a 180° rotation for each 20 mm field of view. The sample was positioned at a distance of 1000 mm from the camera to take advantage of the propagation phase contrast in order to distinguish between tissue types [28]. Phase was retrieved using the method of Paganin *et al*. [29] to improve contrast. Three-dimensional volumes were reconstructed using an in-house high-speed filtered back projection reconstruction algorithm [30]. Three-dimensional datasets resulting from the reconstruction were rendered using commercial software (Avizo, France). The volume of the papillae was measured using the thickness option of the BoneJ plugin in Fiji software (Wikimedia Foundation Inc., USA).

### 2.3. Three-dimensional reconstruction of the equine foot and measurement of the hoof dorsal curvature

Individual hooves were scanned using a Phoenix v|tome|x m industrial scanner (GE, Germany). A maximum X-ray energy of 125 kV, 320 µA current and a 0.5 mm thick copper filter was used to scan each sample, consisting of 2160 projection images, with a detector exposure time of 333 ms, acquired over a 360° rotation. The magnification and spatial resolution achieved were ×1.82 and 120 µm respectively. Data were reconstructed for visualization using datos|x software and were visualized using VGStudio MAX 2.2 (GE, Germany). The average dorsal curvature of the hoof was estimated from a ventro-dorsal sagittal section using Fiji (electronic supplementary material, appendix SM.1).

### 2.4. Histology

Formalin-fixed biopsies were embedded in wax oriented and sectioned every 8 µm. The haematoxylin and eosin (H&E) staining was performed to enable measurement of cell size. For immunostainings, sections were deparaffinized in xylene, rehydrated and incubated in a 10% trisodium citrate buffer (pH6) solution at 60°C for 10 min for antigens retrieval. Blocking solutions, i.e. 5%FBS/PBS (S1) for immunohistochemistry or 2%BSA/PBS (S2) for immunofluorescence, were used for 30 min prior to incubating the primary antibodies for 1 h (Ki-67, 1/100 dilution in S1, Abcam, UK; p63, 1/500 dilution in S1, Gene Text, USA; K14, 1/50 dilution in S2, Abcam, UK; K10, 1/300 dilution in S2, CUSABIO, USA). For immunohistochemistry, the final step was performed using the Novolink Polymer Detection System (Leica Biosystems, UK). For immunofluorescence, Alexa-fluor 594 or Alexa-fluor 488 (Molecular Probes, UK) secondary antibodies were used (1/1000 dilution in S2) for 30 min and mounted using VectorShield (Sigma, UK). DNA fragmentation was determined using ApopTag following the manufacturer's instructions (Sigma, UK). A 0.5% methyl green in a 0.1 M sodium acetate (pH4) solution (Sigma, UK) was used to counterstain the nucleus of non-apoptotic cells. Slides were finally washed in 100% *N*-butanol prior to DPX-mounting (Sigma, UK).

### 2.5. Isolation, pluripotency and immunofluorescence of progenitor keratinocyte cells

Incisions were made dorsally in the coronary band region allowing the proximal hoof wall to be lifted to expose the underlying soft tissue which was then cut into approximately 0.1 cm^3^ pieces and transferred into a 0.25% trypsin–EDTA (Gibco, UK) solution for 18 h. Epidermal tissues containing progenitor keratinocytes were then transferred into a 0.1% collagenase type-1/1% BSA/DMEM (Sigma/Gibco, UK) solution for 2 h at 37°C. Single keratinocytes were obtained by passing the solution through a 40 µm cell strainer (Falcon, UK) prior to centrifugation at 300*g* for 5 min. Cells were re-suspended in 10% FBS-DMEM-F12 (Gibco) and plated at a density of 5 × 10^3^ cells cm^−2^ on collagen-I (Sigma, UK) pre-coated culture flasks. Cells were passaged using trypsin when reaching 80% confluence. The osteogenic differentiation was performed using the StemPro^®^ Osteogenesis Differentiation Kit (Gibco, UK) following the manufacturer's instruction. Cells were then fixed in 4% PFA/PBS for 10 min and stained with 1% Alizarin Red solution (pH 4.2) for 10 min. The proportion of osteogenic cells was assessed using light microscopy. For immunofluorescence staining, cells were fixed for 10 min in 4% PFA/PBS prior to be incubated in 0.15% (w/v) saponin/PBS solution (LLC, France) for 1 h. The S2 blocking solution (see § 2.4.) was used for 1 h prior to adding primary antibodies for 1 h (CD44, 1/200 dilution; Ki-67, 1/250 dilution; K14, 1/200 dilution; all from Abcam and reconstituted in S2). Secondary antibody Alexa-fluor 594 or Alexa-fluor 488 (Molecular Probes, UK) were used at a dilution of 1 : 1000 in S2 for 30 min. Fluorescence intensities were measured using Image Pro (Media Cybernetics Inc., USA).

### 2.6. Field study

One hundred and twenty-nine horses from 12 different yards in the southeast of England were evaluated from 19 September 2016 to 3 October 2016. Eligibility criteria for inclusion in the study included an age greater than 5 years and height under 144 cm with shoes. All horses were healthy at the time of evaluation and none had a history of laminitis/hoof conditions, pre-existing health conditions or were treated for pituitary pars intermedia dysfunction. There were no selection criteria on breed, sex, management or exercise regime. Hoof diameters were measured using a 40 cm ruler across the widest part of the hoof. Horses were walked onto an electronic Horse Weight^®^ bridge and the mass recorded to the nearest kilogram. General adiposity was assessed by body condition scoring (BCS) using the Henneke nine-point scale [31,32]. Lateral hoof photographs were taken for both forefeet with a wooden bar placed behind the heel bulbs. A 40 cm ruler was then placed at the widest aspect of the lateral hoof wall in parallel with the wooden bar to ensure no obliquity in the image. A 15 mm diameter blood tube lid was put on top of the ruler in contact with the lateral hoof wall to act as a scale to determine the parallax. A camera (Nikon Coolpix L330, UK) was placed at the end of the ruler and the photo taken. Images were imported into Fiji for average dorsal wall curvature calculation (electronic supplementary material, appendix SM.1).

### 2.7. Statistical analysis

All statistical analysis and linear regressions were performed using Prism6 (GraphPad, USA). *p*-Values less than 0.05 were taken as statistically significant.

## 3. Results

### 3.1. Visual assessment of a dorsally curved hoof

**Figure 1.**
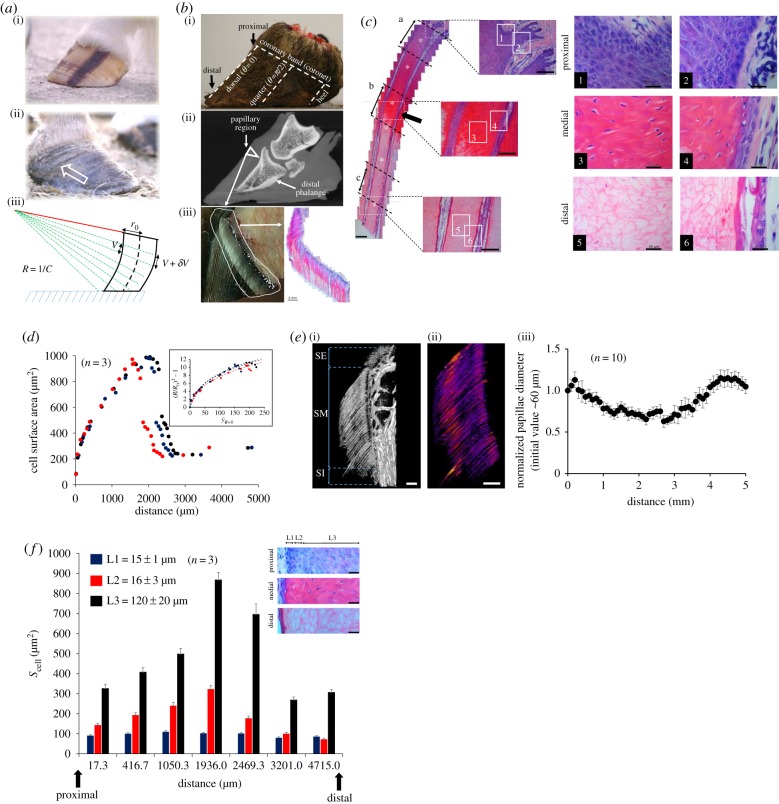
(*a*(i,ii)) Visual characteristics of straight and dorsally curved hooves linked to a differential growth across the coronary band. The arrow points to a ‘diverging growth band’ visible with a naked eye. (*a*(iii)) Based on these observations, a simple geometric model can be inferred to describe how the dorsal curvature of the hoof can appear as a result of a diverging growth from the coronary region. (*b*(i)) Basic anatomical nomenclatures of the equids foot, given the radial symmetry of the hoof an angular notation involving the parameter ‘*θ*’ is used to describe the location at the coronary band. (*b*(ii)) Location of the papillary region with regard to the distal phalange using an X-ray picture of the equid foot. (*b*(iii), left) A dissection of the papillary region demonstrates that the papillary region is also where the epithelium changes its state from being a soft tissue to a hard one. (*b*(iii), right) The papillary region can be immunohistochemically stained using H&E demonstrating the presence of papillae namely soft digit structure (scale bar, 2 mm). (*c*) A magnification of a longitudinal section of papilla labelled with H&E demonstrates the different regions involved in cell differentiation and the hoof synthesis including the blue proximal region, the red cytosolic region and the white region where remnant structures are devoid of cytosol and nuclei (*n* = 3 and scale bar, 200 µm). The black arrow points to a region where the borders of the papillae seem to join. A magnification of interpapillary regions was carried out (scale bar, 100 µm). The numbered squares refer to magnified regions on the right (scale bar, 20 µm). The letters a, b and c refer to proximal, medial and distal regions labelled with K10 and K14 (figure 2*d*). The stars represent the regions where the size of cells, along the axis perpendicular to the papilla, were measured (see (*f*) below). (*d*) Measure of the cell sizes as a function of the distance in the direction of growth. The three colours used (black, dark blue and red) correspond to three different hooves. The inset is the theoretical fit using equation (3.1) (*n* = 3). (*e*(i)) High-intensity X-ray imaging (synchrotron) of horse papillae (*n* = 1) showing three sub-regions of the interpapillary region including the stratum externum (SE), the stratum medium (SM) and the stratum internum (SI). The SM is the papillary sub-region from where the bulk of the hoof is synthesized (scale bar: 2 mm). (*e*(ii)) A three-dimensional reconstruction of the SM sub-region permits measurement of and colour coding the diameter of papillae where the red colour is indicative of a larger diameter as opposed to the blue colour (scale bar: 2 mm). (*e*(iii)) A selection of 10 papillae (*N* = 10) demonstrates that the average diameter of a papilla changes along its longitudinal axis and that a reduction in its diameter is associated with an increase in cell size in the same region (figure 1*d*). Note that the region where the average diameter of papillae is the smallest (distance approx. 2 mm) is also where the borders of the papillae seem to join, see black arrow in *c*. (*f*) Surface area of cells measured along the axis perpendicular to the direction of the papillae at different positions marked by a white star ‘*’ (*c*). Perpendicular to the direction of growth the cell surface area in the interpapillary space changes over a thickness corresponding to two cell layers close to the papillae (L1 and L2). In the L3 region, the interpapillary cells or remnant structures (after the transition) have a homogeneous size that is a function of their progression in the interpapillary space. The error bars correspond to the standard deviation of the cell surface area. Note that L1 + L2 + L3 represents only half the interpapillary transverse length of the interpapillary space. Consequently, it is worth noting that the position of the transition is independent of the surface area of cells. This observation suggests that if the temporal evolution of the size of cells is constrained by the interpapillary pressure, this pressure is not involved in the transition observed. Said differently, the position of the transition is ‘timed’ by the biology of the differentiation of keratinocytes. (‘*n*’ describes the number of hooves used for measurements and ‘*N*’ the number of papillae used).

The presence of a dorsal curvature (a.k.a. Aladdin's slipper shape, figure 1*a*) is indicative in equids, of a past or present pathology. Provided a more or less circular hoof a simple geometric argument suggests that such dorsal curvature is possible because the heel and dorsal regions do not grow at the same rate. This argument is further intuitively confirmed by the occurrence of diverging growth bands on the hoof capsule from the dorsal to the heel regions (see arrow in figure 1*a*), which underlines a differential growth between these regions. To better understand the occurrence of diverging growth bands, a thorough investigation of the initial stages of hoof growth was completed.

**Figure 2.**
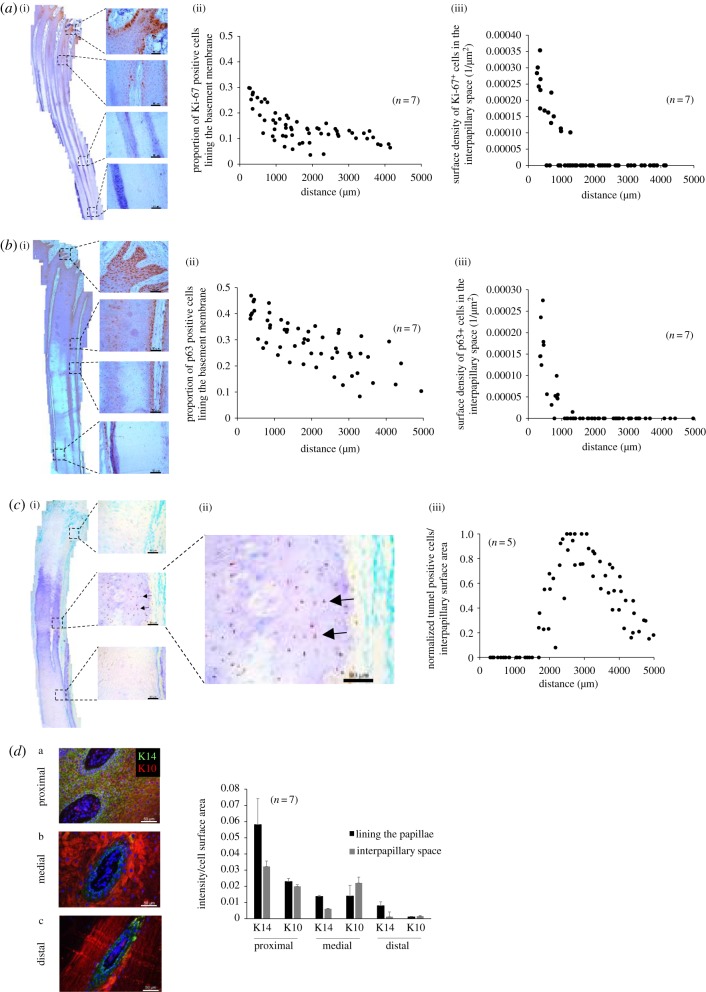
(*a*(i)) Immunohistochemical staining of Ki-67 on a longitudinal section of papillae (scale bar, 50 µm). (*a*(ii)) Proportion of Ki-67 positive cells forming the basement membrane of the papilla as a function of the length of the papilla. (*a*(iii)) Surface density of Ki-67 positive cells in the interpapillary space as a function of the proximo-distal length namely the distance in the direction of hoof growth. (*b*(i)) Immunohistochemical staining of p63 on a longitudinal section of papillae (scale bar, 50 µm). (*b*(ii)) Proportion of p63 positive cells forming the basement membrane of the papilla as a function of the length of the papilla. (*b*(iii)) Surface density of p63 positive cells in the interpapillary space as a function of the proximo-distal length namely the distance in the direction of hoof growth. (*c*(i)) DNA fragmentation measured via TUNEL assay as a function of the distance in the direction of growth (scale bar, 50 µm) the arrows point toward cell showing DNA fragmentation. (*c*(ii)) Magnification of the interpapillary space showing brown nuclei characteristic of DNA laddering. (*c*(iii)) The surface density of TUNEL positive cells was determined, as in (*a*,*b*), as a function of the length of the papillae and normalized by its highest value to determine the interpapillary region where cell death is prominent. The figure demonstrates that this distance (approx. 2 mm) is also where the transition occurs (figure 1*d*) or where the papillary diameter is minimal (figure 1*e*). Note that the interpapillary space includes all cells even those close to the edge of the papilla. (*d*) Surface intensity of K14 and K10 expressed in the basement membrane of papillae and in the interpapillary space (scale bar, 50 µm). Note that the interpapillary space includes all cells even those close to the edge of the papilla (‘*n*’: number of hooves used for measurements).

### 3.2. Biology of the early stages of hoof growth

The synthesis of the hoof capsule starts from the coronet, i.e. the papillary region, and upon dissection and H&E staining of this region dorsally, soft digit structures a.k.a. papillae became visible (figure 1*b*). Between papillae, it was possible to differentiate three interpapillary regions (figure 1*c*). The blue ‘proximal nuclear region’, comprising cells with little cytoplasm and where the nucleus occupies most of the cellular volume. The red ‘cytosolic region’, in which the cytoplasmic volume of cells enlarges prior to its dissipation under the form of a ‘transition’, after which there is no visible cytoplasm nor nucleus but only remains that are expected to be keratin proteins as the hoof is a keratinized tissue. While the location of the ‘transition’ within the interpapillary space did vary slightly between hooves (figure 1*d*), the near-linear rate of cytoplasmic accumulation before the transition ($R^{2}=0.981\pm 0.008$) and cell size at the transition ($S_{\mathrm{cell}}=980\pm 9\;\mu m^{2}$) were similar between hooves, suggesting that the rate of cytoplasmic volume accumulation over time is proportional to the surface area of the cells. Note that within this transition region, the borders of the papillae seemed to join (black arrow figure 1*c*), which may suggest the presence of a pressure within the interpapillary space, possibly linked to the ability of cells to increase their size via accumulation of cytosolic material (figure 1*d*). To relate the changes in the cell surface area (figure 1*d*) to any concomitant variation in the physical appearance of papillae, high energy X-rays were used to reconstruct a three-dimensional image of the papilla in the dorsal region (figure 1*e*(i)). This technique allowed us to extract the papillae from the stratum medium involved in the synthesis of the hoof capsule (figure 1*e*(ii)) and measure their diameter along their proximo-distal axis, i.e. along the axis of hoof growth (figure 1*e*(iii)). The result confirmed that the average diameter of the papilla decreases to become minimal at a typical distance of approximately 2 mm that corresponds to the position of the transition (figure 1*d*).

Perpendicular to the direction of growth, the cells were visibly smaller close to the papillae in a region corresponding to approximately three cell-thickness (figure 1*f*). This last result suggests that perpendicular to the direction of hoof growth the size of cells is relatively homogeneous within the interpapillary space.

Regarding key molecular determinants, Ki-67 (proliferation marker, figure 2*a*) [33], p63 (organization of the epithelium structure, figure 2*b*) [34], DNA fragmentation via TUNEL assay (dead tissue formation, figure 2*c*) [35], K14 and K10 (differentiation markers of the epithelium, figure 2*d*) [36] were labelled and measured. Ki-67 and p63 were expressed in cells lining the basement membrane of the papillae (i.e. the cells forming the papillae itself) and the interpapillary space and their expression decreased distally. However, by considering the variations in the cell surface area (figure 1*d*), the probabilities that cells were still expressing Ki-67 or p63 in the interpapillary space were at most approximately 1% of what was seen in the basement membrane. This result suggests that the proliferation process is negligible in the interpapillary space. Interestingly, although Ki-67 expression was visible over the entire length of the papillae (figure 2*a*(ii)), cells emerged in the interpapillary space only proximally (figure 2*a*(i)) suggesting that cells from the papillae move in the opposite direction of the hoof growth prior to entering in the papillary space. This counter-flow mechanism can be potentially rationalized taking into consideration the interpapillary pressure linked to the changes in cell volume applying a normal pressure against the surface of the papillae (electronic supplementary material, appendix SM.2). DNA fragmentation became clearly visible in the transition region (distance approx. 2 mm, figure 2*c*) and peaked when cell size reached minimal value after the transition (distance approx. 3 mm, figure 2*c*). The keratinocyte progenitor stem cell marker K14 existed at a much higher expression proximally compared to K10, a later stage marker of keratinocyte differentiation (figure 2*d*); and both expressions decreased from the proximal to the distal locations.

Hoof synthesis needs to be seen as a dynamical process and the remarkable changes in cell size as a function of their progression in the interpapillary space, concomitant with the variations in the diameter of papillae, are indicative that physical stresses linked to changes in pressure may be at play to initiate and synchronize the early stage of hoof morphogenesis (electronic supplementary material, appendix SM.2). This pressure would be required in any case for the hoof to grow to balance the weight of the animal and the adhesion of the capsule on the distal phalange. Furthermore, the near-constant cell surface area of transverse cells when the cells move along the direction of growth suggests that a one-dimensional model as a leading approximation is potentially valid to describe the growth stress.

### 3.3. Physics of the early stages of hoof growth

Histological pictures represent a steady-state of the early stage of hoof morphogenesis. To model cell volume changes anywhere at the coronet level, we note by, θ, the angular position (figure 1*b*) by, $R_{\theta }(y)$, the average radius of cells (data from figure 1*d* correspond to $R_{\theta =0}(y)$) and by, $R_{T}(y)$, the biological target radius that cells would have if no physical stresses were involved. In this context, a physical interpapillary pressure can be defined by $P_{\theta }(y)∼K(1-R_{T}^{3}(y)/R_{\theta }^{3}(y))$ that results from the mismatch between the target and real cell sizes, where $K∼10^{3}\mathrm{Pa}$ is the typical elastic modulus of keratinocytes cells [25,26]. However, interpapillary cells have to share the limited interpapillary space and, as a result, the real cell size is also a function of the local amount of cells present in the interpapillary space. Neglecting cell division in the interpapillary space (figure 2*a*) and assuming that the interpapillary space volume does not depend on the angular positioning, by noting $\left(N_{\theta }\right)$ the number of cells in the interpapillary space, the conservation of the interpapillary volume implies that $(N_{\theta }){\left(R_{\theta }\right)}^{3}$ is constant. As a result, variations in the interpapillary pressure are possible if more cells are present in the interpapillary space. By assuming that the differentiation process is independent of the physical stresses present in the interpapillary space (figure 1*f*), it is possible to compare the difference in pressures between two angular positions by determining the number of cells that are present in the interpapillary space at these locations. In the remaining text one shall note, ${\bar{N}}_{\theta }=N_{\theta }/{\left(N_{\theta }\right)}_{0}$, where the variable ${\left(N_{\theta }\right)}_{0}$ is a normalization constant linked at an ‘initial state’ to be defined.

The variation of the cell size along the *y*-axis can now be addressed making use of: (i) the balance of stresses $-dP_{\theta }/dy∼\lambda v_{\theta }/2R_{\theta }$ where λ is a drag constant, $v_{\theta }=2R_{\theta }/\tau_{0}$ the velocity of the cell and $\tau_{0}$ the typical proliferation time of keratinocyte progenitor cells dividing from the proximal part of the papillae; (ii) a rate of volume change per unit of time for the target size that is proportional to the surface area of the cell, or equivalently $dR_{T}/dt∼v_{m}$ where *v*_m_ is the proportionality constant representing the rate of inward flow of mass across the membrane of cells and; (iii) a change in the target size as a function of the position in the interpapillary space written as, $y∼\int 2R_{T}\;dt$. Further assuming that when cells enter the interpapillary space their target and real sizes are the same and is constant whatever the angular position, i.e. $R_{\theta }(y=0)=R_{0}$; by noting $v_{0}=2R_{0}/\tau_{0}$ the cellular proliferation rate and, $\bar{y}=y/2R_{0}$, the set of relations allows one to determine the following rate of growth:

3.1 $$\begin{array}{rl} R_{\theta }/R_{0} & ∼v_{\theta }/v_{0}\\ & ∼\sqrt{1+4v_{m}/v_{0}\times (\bar{y}-1)}/{\left(1+(\lambda v_{0}/K)\times (\bar{y}-1)/{\bar{N}}_{\theta }\right)}^{1/3}. \end{array}$$

Concentrating on the dorsal region and assuming the histological pictures as being initial states, i.e. ${\bar{N}}_{\theta =0}=1$, a nonlinear regression against the data plotted in figure 1*d* provides $4v_{m}/v_{0}∼0.32\pm 0.03$ and $\lambda v_{0}/K∼0.059\pm 0.006$, with (*R*_adj_)^2^ greater than 0.98 for each fit prior to the transition (inset figure 1*d*). From the numerical constants, the typical hoof growth rate can be determined. Assuming that the growth rate is driven by the variation in cell size prior to the transition located at the position ${\bar{y}}_{c}∼199\pm 26$ (figure 1*d*), by using a nominal cell size $R_{0}∼5.14\pm 0.07\;\mu m$ (figure 1*d*) and a typical proliferation time $\tau_{0}∼10\;h$ one finds a theoretical growth rate based solely on the changes in the cells' morphology that is ${\left(v_{\theta =0}\right)}_{c}∼0.17\;\mathrm{mm}\;\mathrm{da}y^{-1}$, which is the order of magnitude of the hoof growth rate in different ungulate species (table 1; note that ${(v\theta =0)}_{c}∼0.3\;\mathrm{mm}\;\mathrm{da}y^{-1}$ with $\tau_{0}∼5.6\;h$). Thus, the position of the transition seems to match the soft-to-hard transition namely the location at which point the hoof becomes a solid and reaches its steady growth rate.

**Table 1.** Hoof growth rate in different ungulate species.

animal	growth rate (mm day^−1^)	refs
horse	0.2–0.3	[24]
sheep	0.1–0.2	[37]
deer	0.1–0.2	[38]
cow	0.1–0.3	[39,40]

By virtue of the balance of stresses, the interpapillary pressure can also be determined under the form:

3.2 $$P_{\theta }∼{\left(P_{\theta }\right)}_{0}-\lambda v_{0}(\bar{y}-1)/{\bar{N}}_{\theta },$$

where, ${\left(P_{\theta }\right)}_{0},$ is the interpapillary proximal pressure, i.e. for $\bar{y}=1$, generated by the papillae and with a magnitude that can be estimated using figure 2*a*, i.e. when ${\bar{N}}_{\theta =0}=1$, leading to ${\left(P_{\theta =0}\right)}_{0}∼1.9\times 10^{5}\;\mathrm{Pa}\pm 4.9\times 10^{4}\;\mathrm{Pa}$ (electronic supplementary material, appendix SM.3). This magnitude that has an order of magnitude similar to the values found in the field study (see thereafter) allows one, by using equation (3.2) and figure 1*d*, to estimate that the pressure at transition, ${\left(P_{\theta =0}\right)}_{c},$ is: ${\left(P_{\theta =0}\right)}_{c}∼0.94\times {\left(P_{\theta =0}\right)}_{0}∼1.8\times 10^{5}\;\mathrm{Pa}$.

As beyond the transition there is no possibility for cells to actively generate any further growth, the pressure at the transition, ${\left(P_{\theta }\right)}_{c}$, should correspond to the growth stress of the hoof. In this instance, it can be assumed that the process of cornification/horn formation, i.e. the transition, is also a means of balancing external stresses, and the presence of the transition is probably linked to the reorganization of keratin filaments from dead cells into larger bundles under pressure so that the hoof is synthesized to match its function as a weight-bearing element.

### 3.4. The soft-to-hard transition

The reason for which the transition in size corresponds to the formation of the hard horn can be understood as follows. Firstly, let us assume that cells have to be in a certain biological state when cell death is ongoing to collapse, as observed in figure 1*c,d*. In this context, one assumes that the transition is driven by the pressure involved at the transition. Secondly, let us assume also that prior to collapsing, each non-aggregated keratin filament occupies a volume *V*_0_ within living cells that is their individual degree of freedom. Thus upon cell death, by neglecting the binding energy between keratins, the entropy gained by forcing the formation of one thick fibril containing ‘n’ non-aggregated intermediate filaments is, $T\Delta S∼-nk_{B}T\ln(n),$ where $k_{B}T$ is the thermal energy. In parallel, this aggregation releases the mechanical energy $-{\left(P_{\theta =0}\right)}_{c}\Delta V∼{\left(P_{\theta =0}\right)}_{c}V_{0}(n-1)$. Equating both relations gives: $\ln(n)∼{\left(P_{\theta =0}\right)}_{c}V_{0}/k_{B}T\times (1-1/n)$. Assuming a typical keratin dimer radius of approximately 14 nm [41] at room temperature one finds *n* approximately 20. As the bending stiffness of filaments is proportional to the fourth power of their radius, the mechanical resilience of the keratin bundle gained through this reorganization would be approximately 20^4^ or equivalently approximately 10^5^. This means that the persistence length determined when the bending energy equals the thermal energy should be increased by a similar factor [42]. Let us assume a persistence length of keratin filament approximately 0.5 µm in two-dimensional cell culture conditions [43], then the reorganization of keratin filaments would increase this value to approximately 80 cm making a hoof with a typical dimension approximately 10 cm a solid structure. Finally, the reorganization of keratin filaments can also explain the approximately 10^5^ order of magnitude difference between the elastic moduli of keratinocytes and the solid keratinized hoof capsule.

Passed this transition, a mechanical description of the hoof capsule treated as a solid can provide a better understanding of the underlying bio-physical mechanisms regulating hoof shape.

### 3.5. Stresses balance in the hoof capsule

Straight or curved hooves redistribute the different loads across the coronary band. As the transition is supposed to balance the external load, there is no residual shear stress present in the solid hoof capsule. Let us assume a circular hoof capsule of constant radius *r*_0_ that is curved dorsally modelled as a two-dimensional object of constant thickness *e* and of longitudinal length $Z_{\theta }$, where θ is the angular position on the coronet as defined above. In this context, it is possible to define a reduced variable, $CZ_{\theta }$, where *C* is the dorsal curvature (i.e. for θ=0) that we shall assume constant and much smaller than any other spatial dimensions describing the hoof. The local growth stress, $e{\left(P_{\theta }\right)}_{c}$, defined as the growth force applied per unit of length of the coronet needs to balance the adhesion of the hoof capsule on the distal phalange and the ground reaction to the weight, which using equation (3.2) can be written as:

3.3 $$e[{\left(P_{\theta }\right)}_{0}-\lambda v_{0}({\left({\bar{y}}_{\theta }\right)}_{c}-1)/{\bar{N}}_{\theta }]=f_{\mathrm{adh}}^{0}{\left(v_{\theta }\right)}_{c}\;\;Z_{\theta }+\rho ge\Omega (CZ_{\theta }).$$

In the r.h.s., the first term is the adhesion stress that is proportional to the angular growth rate of the hoof ${\left(v_{\theta }\right)}_{c}$ and the constant $f_{\mathrm{adh}}^{0}$ that characterizes the adhesion of the hoof [44]; and the second term is the component of the ground reaction to the weight applied on the surface of the hoof capsule (in-plane description). Thus, $CZ_{\theta }\ll 1$, *g* is the gravity constant and *ρ* the mass of the animal per surface area of contact between the animal's foot and the ground.

### 3.6. Physical condition required for the straight hoof capsule

As equation (3.3) describes the set of hoof shapes, the ideal case of a straight hoof can now be discussed. Let us assume that ${\left(P_{\theta }\right)}_{0}$ is constant (electronic supplementary material, appendix SM.3) and a constant growth rate whatever the angular position considered. As a straight hoof implies $CZ_{\theta }=0$, the angular variation in the growth stress in this case is necessarily linked to the asymmetry of equids' foot via the adhesion term, in turn imposing a physical condition on ${\left(N_{\theta }\right)}_{0}$. To determine ${\left(N_{\theta }\right)}_{0}$, let us consider as reference the dorsal region as far as the amount of interpapillary cells is concerned, i.e. ${\bar{N}}_{\theta }=N_{\theta }/{\left(N_{\theta =0}\right)}_{0}$; and consider the ‘initial state’ as being the ideal straight hoof, namely ${\left({\bar{N}}_{\theta }\right)}_{0}={\left(N_{\theta }\right)}_{0}/{\left(N_{\theta =0}\right)}_{0}$ where the subscript ‘0’ refers to the straight hoof. Using equation (3.3) in the dorsal region and for any angular positioning, the difference in the balances of stresses gives: $e\lambda v_{0}[\left({\left({\bar{y}}_{\theta }\right)}_{c}-1)/{\left({\bar{N}}_{\theta }\right)}_{0}-({\left({\bar{y}}_{\theta =0}\right)}_{c}-1\right)]=f_{\mathrm{adh}}^{0}{\left(v_{\theta =0}\right)}_{c}Z_{\theta =0}(1-\gamma_{\theta })$, where $\gamma_{\theta }=Z_{\theta }/Z_{\theta =0}$, which depends on two variables ${\left({\bar{y}}_{\theta }\right)}_{c}$ and ${\left({\bar{N}}_{\theta }\right)}_{0}$. However, a relation between these variables exists if one assumes that the cells need to be in a certain biological state after a given biological time, $t_{B}={\bar{t}}_{B}\times \tau_{0}$, before their volume is allowed to collapse at the transition. In this case, whatever the angular position, the relation is given by: $\int_{1}^{{\left({\bar{y}}_{\theta }\right)}_{c}}(v_{0}/v_{\theta })\;d{\bar{y}}_{\theta }∼{\bar{t}}_{B}$ (${\bar{t}}_{B}∼74$ for ${\left({\bar{N}}_{\theta =0}\right)}_{0}=1$). As a result, any small or moderate changes in the relative amount of cells in the interpapillary space from 1 to ${\left({\bar{N}}_{\theta }\right)}_{0}$ shifts the position of the transition by ${\left({\bar{y}}_{\theta }\right)}_{c}-{\left({\bar{y}}_{\theta =0}\right)}_{c}∼{\left({\bar{y}}_{\theta =0}\right)}_{c}\chi [{\left({\bar{N}}_{\theta }\right)}_{0}-1]$, where χ∼−0.62 (electronic supplementary material, appendix SM.4). Thus, as ${\left({\bar{y}}_{\theta =0}\right)}_{c}\gg 1$, one finds the ideal straight hoof condition: ${\left({\bar{N}}_{\theta }\right)}_{0}∼1/[1+f_{\mathrm{adh}}^{0}{\left(v_{\theta =0}\right)}_{c}Z_{\theta =0}(1-\gamma_{\theta })/e\lambda v_{0}{\left({\bar{y}}_{\theta =0}\right)}_{c}(1-\chi )]$. It is worth noting that any deviation from the latter relation should promote the formation of a curved hoof.

### 3.7. A kinematic point of view for the occurrence of a dorsal curvature

Following figure 1*a*, the gradient in growth rate is key to define the dorsal curvature of a hoof. As equation (3.1) defines the angular growth rate of the hoof it is possible to relate the gradient in growth rate to the dorsal curvature. In order to achieve this, let us consider that ${\left({\bar{N}}_{\theta }\right)}_{0}$ is transformed to ${\bar{N}}_{\theta }=N_{\theta }/N_{\theta =0}$ and assume that $N_{\theta =0}∼{\left(N_{\theta =0}\right)}_{0}$. As the relative growth rate along the coronet is proportional to the hoof curvature under the form $C\theta r_{0}∼{\left(V_{\theta }\right)}_{c}/{\left(V_{\theta =0}\right)}_{c}-1$ (figure 1*a*); by considering the first order in ${\bar{N}}_{\theta }-{\left({\bar{N}}_{\theta }\right)}_{0}$ of equation (3.1) one finds, $C\theta r_{0}∼(\alpha +\beta \chi )({\bar{N}}_{\theta }-{\left({\bar{N}}_{\theta }\right)}_{0})$, where α∼0.31 and β∼0.19 are the first derivatives of the growth rate with regard to ${\bar{N}}_{\theta }$ and ${\left(\bar{y}\theta \right)}_{c}$, respectively, at θ=0 (electronic supplementary material, appendix SM.5). Focusing on the quarter regions (θ=π/2) and using *r*_0_ ∼ 5 cm typically, one finds a numerical slope approximately 2.4 m^−1^.

To validate the model, as the interpapillary space and papillae form a closed system, namely that cells proliferating from the papillae should populate the interpapillary space in due course, the experimental relationship between the ability of cells to proliferate locally from the papillae at the quarter and dorsal regions and the average dorsal curvature of the hoof were determined using equine feet. The average dorsal curvature of the capsule was calculated using a dorsoventral sagittal section of the hoof using µCT scanning. The feet were then dissected to estimate the rates of progenitor cell proliferation from papillae (average proportion of Ki-67 positive cells to total number of cells) at dorsal (θ∼0) and quarter (θ∼π/2) regions as done in figure 2*a*, to estimate $N_{\theta =\pi /2}^{\;}/N_{\theta =0}^{\;}$. In order to validate $N_{\theta =0}∼{\left(N_{\theta =0}\right)}_{0}$, the feet selected were those where the standard deviation in the proportion of Ki-67 positive cells at the dorsal regions was approximately 10% or less from the average value.

**Figure 3.**
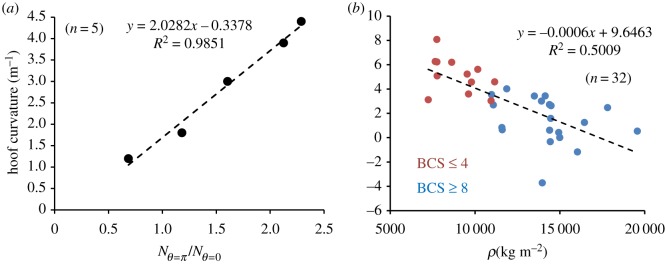
(*a*) Average dorsal hoof curvatures as a function of the relative number of Ki-67 positive cells in the basement membrane of papilla at the quarter and dorsal regions. (*b*) Linear trend between the average dorsal hoof curvatures (measured as described in ‘Material and methods' and further developed in electronic supplementary material, SM.1) and the mass per solar surface area for extreme BCS scores (*p* < 10^−4^). (‘*n*’: number of hooves used for measurements).

Figure 3*a* shows a linear trend with a slope approximately 2.0 m^−1^ between the dorsal curvature of hooves as a function of $N_{\theta =\pi /2}^{\;}/N_{\theta =0}^{\;}$, with a magnitude similar to the predicted value. Using the slope and constant terms from the fit figure 3*a*, it can be estimated that the curvature is null if ${\left({\bar{N}}_{\theta =\pi /2}\right)}_{0}∼0.16$, leading to $f_{\mathrm{adh}}^{0}{\left(v_{\theta =0}\right)}_{c}∼2.9\times 10^{4}\;\mathrm{Pa}$ by using typically $\gamma_{\theta =\pi /2}∼2/3$ [45], $Z_{\theta =0}∼10\;\mathrm{cm}$ and *e* approximately 1 cm.

As a result, curved or ‘dished’ hooves result from an excess of cell proliferation from the quarter regions. To what extent is physics key in this process can only be clarified by using the full stresses balance.

### 3.8. Physics of hoof shape: impact of chronic changes in the horse weights

To further the knowledge of hoof growth $\Omega \;(CZ_{\theta })$ was estimated using the hoof symmetry: firstly by considering the mediolateral symmetry of the hoof leading to $\left(\partial \Omega \;/\partial Z_{\theta }\right){|}_{Z_{\theta =0}}=0$; and secondly, as the heel is shorter than the toe and that the magnitude of projection of the ground reaction stress decreases from the quarters to the toe, $\left(\partial \Omega \;/\partial Z_{\theta }\right){|}_{Z_{\theta }\neq Z_{\theta =0}}<0$. In this context, a second-order development around $CZ_{\theta =0}$ can be envisaged, $\Omega \;(CZ_{\theta })∼\Omega \;(CZ_{\theta =0})+a/2\times {\left(CZ_{\theta }-CZ_{\theta =0}\right)}^{2}$, where *a* > 0 is a constant. Therefore, using ${\left(v_{\theta =\pi /2}\right)}_{c}/{\left(v_{\theta =0}\right)}_{c}-1∼Cr_{0}\pi /2$, the difference in the balances of stresses between the quarter and toe regions can be determined: $e\delta P_{c}∼-f_{\mathrm{adh}}^{0}{\left(v_{\theta =0}\right)}_{c}\Delta Z+f_{\mathrm{adh}}^{0}{\left(v_{\theta =0}\right)}_{c}Cr_{0}Z_{\theta =\pi /2}\pi /2+a\rho geC^{2}{\left(\Delta Z\right)}^{2}/2$, where $\delta P_{c}={\left(P_{\theta =\pi /2}\right)}_{c}-{\left(P_{\theta =0}\right)}_{c}$ and $\Delta Z=Z_{\theta =0}-Z_{\theta =\pi /2}>0$. Finally, noting $\bar{C}=CZ_{\theta =0}$ and $1/\rho_{0}=\mathrm{age}{\left(1-\gamma_{\theta =\pi /2}\right)}^{2}/f_{\mathrm{adh}}^{0}{\left(v_{\theta =0}\right)}_{c}r_{0}\gamma \pi$, the physical solution for the dorsal curvature at the lowest order in ρ is:

3.4 $$\begin{array}{rl} & \bar{C}∼{\Omega \;}_{0}-{\Omega \;}_{0}^{2}\rho /\rho_{0}\\ & {\Omega \;}_{0}=\frac{2Z_{\theta =0}(1-\gamma_{\theta =\pi /2})}{\pi r_{0}\gamma_{\theta =\pi /2}}\left[1+\frac{e\delta P_{c}}{\;f_{\mathrm{adh}}^{0}{\left(v_{\theta =0}\right)}_{c}Z_{\theta =0}(1-\gamma_{\theta =\pi /2})}\right]. \end{array}$$

Equation (3.4) stipulates that the maintenance of a straight hoof, i.e. $\bar{C}=0$, is possible if the gradient in growth stress matches exactly the asymmetrical design of the hoof, i.e. when ${\Omega \;}_{0}∼0$ or equivalently that ${\bar{N}}_{\theta }={\left({\bar{N}}_{\theta }\right)}_{0}$; and/or that the mass per unit of surface area of hoof is $\rho ∼\rho_{0}/{\Omega \;}_{0}$. It is worth noting that in this latter case and by virtue of the definition of $\rho_{0}$, the ratio between the adhesion and ground reaction stresses must verify: $f_{\mathrm{adh}}^{0}{\left(v_{\theta =0}\right)}_{c}/ag\rho ∼e{\Omega \;}_{0}{\left(1-\gamma_{\theta =\pi /2}\right)}^{2}/r_{0}\gamma_{\theta =\pi /2}\pi$; namely that to maintain a straight hoof the variation in the hoof growth rate needs to be related to the variation of the horse mass under the form: $\delta {\left(v_{\theta =0}\right)}_{c}∼+\delta \rho$.

In order to address these points, 129 ponies were selected and their body condition score (BCS) assessed using the Henneke nine-point scale [31,32], with a score of 1 being an emaciated/unwell horse and a score of 9 being an overweight/obese horse. As scores ranged between 3 and 8, three broad BCS categories were defined as underweight, normal and overweight linked to, respectively, BCS ≤ 4 (*n* = 12), 4 < BCS ≤ 7 (*n* = 187) and BCS ≥ 8 (*n* = 20) where ‘*n*’ is the number of feet. The stress linked to the weight was estimated on the forefeet knowing the animals' mass and foot circumference. The hoof dorsal curvature was calculated using a lateral picture of the hoof. Given that the weight appears as a second-order term in the quadratic equation related to the stresses balance, only extreme cases were plotted, i.e. BCS less than or equal to 4 and BCS greater than or equal to 8, demonstrating a negative relationship between the stress linked to weight and hoof curvature (figure 3*b*, *p*-value less than or equal to 10^−4^).

The concordance between the theory and measurements can be underlined from the fit figure 3*b*. In this context, one can estimate that a null curvature occurs for $\rho_{0}/{\Omega \;}_{0}∼1.7\times 10^{4}\;\mathrm{kg}\;m^{-2}$, which by considering the constant term of the fit corresponding to ${\Omega \;}_{0}/Z_{\theta =0}∼9.6m^{-1}$ and assuming typically that $Z_{\theta =0}∼10\;\mathrm{cm}$ allows one to estimate firstly ${\Omega \;}_{0}∼0.96$ and secondly a slope $∼-5\times 10^{-4}m\;kg^{-1}$, which is close to $∼-6\times 10^{-4}m\;kg^{-1}$ experimentally deduced. Note also that by multiplying $\rho_{0}/{\Omega \;}_{0}$ by the gravity constant ($g∼9.8\;m\;s^{-2}$) a magnitude for the ground reaction stress $∼1.6\times 10^{5}\mathrm{Pa}$ can be estimated, which has the same order of magnitude as the growth stress deduced from histology pictures $∼1.8\times 10^{5}\mathrm{Pa}$.

As ${\Omega \;}_{0}$ seemed to be a positive constant one can expect that the angular gradient in the growth stress vanishes in case of extreme BCSs. This point is further suggested as in these conditions and from equation (3.4), ${\Omega \;}_{0}∼2Z_{\theta =0}(1-\gamma_{\theta =\pi /2})/\pi r_{0}\gamma_{\theta =\pi /2}$, that in turn allows one to estimate theoretically ${\Omega \;}_{0}/Z_{\theta =0}∼6.4\;m^{-1}$, which has an order of magnitude similar to the curvature experimentally found $∼9.6\;m^{-1}$. This result suggests that for extreme BCSs the biology of hoof growth does not compensate for the physics associated with the asymmetry of the hoof capsule.

Thus, it is the magnitude of the pressure load linked to the equids’ weight applied onto the distal edge of its hoof that drives the dorsal curvature, which means also that for the equids studied the straight hoof condition, $\delta {\left(v_{\theta =0}\right)}_{c}∼+\delta \rho$, is not fulfilled. This statement can be deduced directly using equation (3.3) by comparing how the relative growth rate changes at the dorsal region as a function of a relative change in the horse mass. In this context, it can be shown that $\delta {\left(v_{\theta =0}\right)}_{c}∼-\delta \rho$ (electronic supplementary material, appendix SM.5). Thus, the straight hoof condition is never fulfilled that, in turn, underlines a central issue regarding the equids' hoof.

## 4. Discussion

In developing countries, owing to the fact that good husbandry and veterinarians are expensive to afford [46], between 70% [47] and 85% [46] of equids have a low BCS (less than 4 on the nine-point Hanneke scale) and chronic pathological conformations of the foot, limb deformities and foot pain are widespread issues [4,46,47]. Given the social and economic importance of working equids, a multi-scale theoretical framework is provided to improve our knowledge of chronic hoof shape variations.

### 4.1. The main anatomical–biological–physical issue regarding hoof growth

Owing to the fact that a three-dimensional histologic resolution at the cellular scale was not feasible and that no specific cellular organizations were observed in the interpapillary space, a minimalist model considering the interpapillary cells as being spherical was used. The concepts applied would not be affected if cells had different geometry as only geometric constants would change. Finally, the coherence between the theory and the experimental data provides a guarantee that this assumption is sound. As a result, the hoof growth rate is principally related to the number of keratinocytes duplicating from the papillae and how well these can swell while differentiating in the interpapillary space. The process whereby soft tissues make hard ones is essential in ungulates as the hoof is a weight-bearing element and the mechanical resilience of the hoof has to be adjusted to the horse weight. This process is in theory possible by the ability of dead soft structures to reorganize single keratin filaments into large connected bundles under pressure. Indeed, the role of the local pressure in the interpapillary space, namely the presence of two opposed pressures applied to differentiating cells that are the growth stress and ground reaction to the weight, seems to mark the main difference between the hoof and the hair follicle. In the hair follicle where no distal stress is present, the keratinization process is continuous [48]. In the hoof, a transition is observed suggesting a precipitation of the keratinization process. Naturally, the presence of the transition does not rule out the importance of how dry the environment is [48,49].

Therefore, provided a more or less constant hoof growth rate, with quarters shorter than the toe and an adhesion stress proportional to the longitudinal length of the hoof capsule, the asymmetry of the horse's foot means that unless the asymmetry is properly adjusted, its quarter regions have a tendency to grow slightly faster when compared to the toe region leading to the occurrence of a dorsal curvature. However, the tendency for a dorsal curvature can be modulated, i.e. rendered invisible or exacerbated, by the weight of the animal. Said differently, in an imaginary world devoid of gravity, equids hooves should curve automatically. To demonstrate this point, we have concentrated on extreme BCSs whereby the impact of gravity/weight becomes physically and mathematically significant. However, we have observed disparities in the dorsal curvature of hooves analysed in equids with normal BCS. These disparities are very likely linked to the different horse genotypes or gene sets involved in hoof growth. Therefore, although physics can describe hoof shape, the underlying biology of hoof growth remains an essential ingredient regarding hoof deformities (see thereafter EMS/ECD cases).

The conclusion deduced from our study regarding the BCS of equids does not imply that overloading an animal excessively over long period of time is necessarily a good thing. On the contrary, it is possible to define a critical loading blocking the dorsal growth of the hoof that can reignite the formation of a dished hoof. Indeed as equids feet are asymmetrical and that the growth stress is homogeneous along the coronet, this critical loading would not block the growth of the quarter regions (as the adhesion stress is lower in these regions). Thus, beyond this critical loading, the formation of a dished hoof should reappear. Numerically, this critical loading can be estimated as being 2.3 times the optimal weight of an animal required to have straight hooves. With regard to the leisure equids used in this study, this would correspond numerically to a loading $∼3.7\times 10^{4}\;\;\mathrm{kg}\;m^{-2}$ (electronic supplementary material, appendix SM.6).

### 4.2. Toward a unification of aetiologies regarding hoof conditions/shapes

Finally, it is also worth noting that the physical parameter that defines the low and high BCS regions is given by $\rho /\rho_{0}∼\rho /{\left(v_{\theta =0}\right)}_{c}$. This means that any increase in the hoof growth rate, which can occur when more cells enter the interpapillary space homogeneously along the coronary band, brought about by a specific genotype or via a biological stimulation irrespective of a change in the animal weight should lead, in physical terms, to a lower BCS. In Western countries, chronic hoof deformities are also associated with the endocrine disorders equine metabolic syndrome (EMS) and pituitary pars intermedia dysfunction (or Cushing's disease or ECD) [50]. Both EMS and ECD are linked to high blood insulin levels. EMS is mostly related to horse obesity [51] and EMS-affected horses demonstrate hyperinsulinaemia [52]. In ECD, high blood cortisol levels are thought to antagonize insulin responses post receptor level [53–55] resulting in high blood insulin levels secreted from the pancreas [56]. As insulin promotes the nuclear translocation of Ki-67 in equids keratinocyte progenitor cells (electronic supplementary material, appendix SM.7), it is worth considering whether hyperinsulinaemia can be involved in promoting a positive dorsal curvature by allowing more cells to enter the interpapillary space. In this context, provided that the proliferation rate is homogeneous across the coronet, the growth rate of the hoof should diverge at the quarter regions when compared to the dorsal part of the hoof leading to dished hooves (electronic supplementary material, appendix SM.8). It is interesting to note, however, that obesity (weight gain) should not be an issue (figure 3*b*).

### 4.3. Summary

Taken together the results provide: (i) an understanding of the bio-physical contradictions between synthesizing a hoof using soft matter, its resulting mechanical properties and foot asymmetry and, as a result; (ii) a potential connection between the different bio-physical aetiologies leading to the occurrence of a dorsally curved hoof, explaining why Aladdin's slippers are ‘natural’ and possible in Equids. Finally, this work underlines also the importance of providing a prescriptive understanding of the physics of animal health, prior to starting any live animal experiments.

## Supplementary Material

Supplementary Materials for Physics of Animal Health: On the mechano-biology of hoof growth and form

## Data accessibility

The datasets generated and analysed during the current study are available from the corresponding authors on request.
