# Supplementary material for: Physics of animal health: on the mechano-biology of hoof growth and form
Source: J R Soc Interface. 2019 Jun 26;16(155):20190214. doi: 10.1098/rsif.2019.0214 (PMC6597769; doi:10.1098/rsif.2019.0214)
Supplement: Supplementary Materials for Physics of Animal Health: On the mechano-biology of hoof growth and form [file rsif20190214supp1.docx]

**Supplementary Materials for**

**Physics of Animal Health: On the mechano-biology of hoof growth and form**

Ramzi Al-Agele^1,6^, Emily Paul^1^, Sophie Taylor^1^, Charlotte Watson^1^, Craig Sturrock^2^, Michael Drakopoulos^3^, Robert C. Atwood^3^, Catrin S. Rutland^1^, Nicola Menzies-Gow^4^, Edd Knowles^4^, Jonathan Elliott^4^, Patricia Harris^5^ and Cyril Rauch^1*^

^1^ School of Veterinary Medicine and Science, University of Nottingham, College Road, Sutton Bonington, LE12 5RD, UK.

^2^ CIPB, Hounsfield Building, University of Nottingham, College Road, Sutton Bonington, LE12 5RD, UK

^3^ BL12, Diamond Light Source Ltd, Diamond House, Harwell Science & Innovation Campus, Didcot, Oxfordshire, OX11 0DE, UK.

^4^ The Royal Veterinary College, Hawkshead Lane, Hatfield, Hertfordshire AL97TA, UK.

^5^ Equine Studies Group, WALTHAM Centre for Pet Nutrition, Melton Mowbray, Leicester LE14 4RT, UK

^6^ Department of Anatomy, College of Veterinary Medicine, University of Diyala, Iraq

^*^***Corresponding authors***: Dr Cyril Rauch. Tel.: +44 (0)115 9516451. Email: [cyril.rauch@nottingham.ac.uk](mailto:cyril.rauch@nottingham.ac.uk).

**SM.0 Basic anatomy and microanatomy of the hoof**

Compared to the human skin for example, the hoof remains an understudied tissue. This section aims at summarizing the state of knowledge concerning the anatomy of the hoof and how it grows as taught in a veterinary curriculum. Parallels and differences with the skin will also be made to underline how complex and different this tissue is. For more information on this vast subject we recommend the reader interested in the hoof topic the following books: GOODY, P. C. 2000. *Horse Anatomy: a pictorial approach to equine structure,* London, J. A. Allen and; DAVIES, H., PHILIP, C. & MERRITT, J. S. 2007. Functional anatomy of the equine digit: determining function from structure. *Equine Podiatry. Saunders Elsevier, St. Louis*.

**Basic anatomy (see Figure SM.0)**

As perissodactyls, or odd-toed ungulates, horses have a strong outer capsule, the hoof, protecting the internal structures of each single-digit foot. This horny structure encloses the distal part of the second phalanx, the distal phalanx and the navicular bone, in addition to connective tissues [[1](#_ENREF_1)].

The proximal part of the hoof joins to the skin at the coronet (or coronary band). The hoof is composed of horn, derived from epidermal tissue which has been keratinised to a varying extent. Horn is largely arranged into a series of parallel microscopic tubules, interconnected by intertubular horn thought to play a substantial role in load-bearing [[2](#_ENREF_2)]. Encasing the palmar/plantar surface of the foot is the sole, which is concave and has a softer and more flexible composition. Connecting the periphery of the sole to the hoof wall is the white line that is derived from the epidermal lamella.

Composed of supple, incompletely keratinised horn, the frog located on the sole is an elastic triangular shaped structure which plays roles in shock absorption and blood circulation. The frog extends inwards to the digital cushion which, being composed of poorly vascularised adipose tissue embedded in a fibroelastic mesh, acts to further enhance the frog’s shock absorbing and blood pumping properties. The digital cushion is segregated from the deep digital flexor tendon (DDFT) by the presence of the distal digital annular ligament. The bones of the equine foot comprise the third phalanx (P3), the second phalanx (P2), and the navicular bone. P3 is the foot’s principal bone, occupying its most distal position, and attaching to the hoof capsule via the lamellar and solar coria. P3 has a very particular shape owing to its function of supporting and stabilising the hoof capsule, and is highly porous due to the prolificacy of nutrient foramina. P2, or short pastern, forms the proximal interphalageal, or pastern, joint with the first phalanx (P1), and the distal interphalangeal, or coffin, joint with P3. Its short, almost cuboidal, composure makes P2 resilient to a broad range of stresses. The navicular, or distal sesamoid bone, is a small, smooth bone located caudal to the distal interphalangeal joint. Coated ventrally in smooth fibrocartilage, it fulfils a pulley-like role, allowing the DDFT to glide smoothly under the distal interphalangeal joint without interference from the other bones. The navicular synovial bursa and distal synovial sheathes further aid the smooth action of the DDFT via the secretion of lubricating synovial fluid.

Along with the DDFT, which descends from the deep digital flexor muscle in the forearm to the flexor surface of P3, the superficial digital flexor tendon (SDFT) forms part of the back tendon pair which permits flexion of the interphalangeal joints. Descending from the superficial digital flexor muscle in the forearm, the SDFT attaches to the proximal surfaces of P1 and P2. Responsible for the extension of the interphalangeal joints is the common digital extensor tendon (CDET). Stemming from the long digital extensor muscle proximal to the knee, the CDET descends the leg dorsally, terminating at the extensor process of P3 with projections into P1 and P2.

While the DDFT and SDFT permit flexion of the foot’s interphalangeal joints and the CDET allows their flexion, the presence of lateral and medial collateral ligaments limits the joints’ adduction and abduction respectively. The collateral ligaments attach to notches on the distal and proximal edges of P1 and P2 correspondingly in the case of those of the proximal interphalangeal joint, and on the distal and proximal edges of P2 and P3 respectively for those of the distal interphalangeal joint. The position of P3 is also maintained by three pairs of chondral ligaments, attaching to the medial and lateral cartilages of P3. The navicular bone is held in place by the navicular suspensory ligaments which anchor to the distal edge of P1, just dorsal to the collateral ligament attachments, and converge at the navicular bone, forming the distal navicular ligament which terminates at P3.

The coria are the richly vascularised and innervated dermal regions lying between and supporting the skeletal structures and the epidermal hoof capsule [[3](#_ENREF_3)]. The coronary corium runs along the proximal edge of the hoof wall, with each hoof wall tubule growing around small, finger-like papillae projecting from the coronary corium which provide nourishment to the proliferative epidermal cells, maintaining hoof growth [[4](#_ENREF_4)]. The solar corium is similar in structure and function to the coronary corium, with papillae enabling the growth of the sole [[4](#_ENREF_4)]. The lamellae of the lamellar corium, form, together with the epidermal lamellae of the inner hoof wall with which they interlock, the suspensory apparatus of the third phalanx, suspending P3 within the hoof capsule. This interlocking provides a large surface area between the epidermis and the dermis for the suspension of P3. Indeed, the lamellar tissue is composed of 550-600 primary epidermal lamellae (PEL), descending the inner surface of the hoof wall in parallel, vertical ridges, each bearing a further 150-200 secondary epidermal lamellae (SEL), which interdigitate with the primary and secondary dermal lamellae (PDL and SDL) respectively [[2](#_ENREF_2)]. *In silico* modelling has shown that this unique hierarchical structure is remarkably resilient to any abrupt mechanical stress that could arise due to locomotion [[5](#_ENREF_5)]. The epidermal and dermal lamellae, is seprated by a surface to which the epidermal cells can adhere through cell:matrix adhesions such as hemidesmosomes [[6](#_ENREF_6)].

**Hoof growth**

The hoof grows thanks to the constant addition of new hoof material to the proximal aspect of the hooves, with proliferative cells found in the epidermal tissues of the coronet and proximal lamellae only [[4](#_ENREF_4)]. At the coronet, the epidermis grows around the small, finger-like projections of the dermis, the dermal papillae, giving the hoof wall its characteristic tubular structure [[6](#_ENREF_6)].

As the hoof wall grows, it must pass the distal phalange smoothly yet without losing adhesion. While this phenomenon was once believed to be permitted by “lamellar flow”, a theory in which cells on the surface of the epidermal lamellae were supposed to proliferate continually to allow the gradual descent of the hoof wall, the discovery that the vast majority of lamellar epidermal cells are non-proliferative lead to the discrediting of this theory [[4](#_ENREF_4), [7](#_ENREF_7)]. Instead, it is now believed that adhesion molecules, such as hemidesmosomes, continually detach and re-attach in a staggered, ratchet-like fashion [[4](#_ENREF_4), [8](#_ENREF_8)]. This it thought to be either through the mediation of matrix metalloproteinase (MMP), which cleaves the hemidesmosome protein integrin where it attaches to the BM, and its inhibitor, tissue inhibitor of metalloproteinase [[4](#_ENREF_4), [9](#_ENREF_9)] or via the slow movement of the capsule which together with the thermal agitation of adhesive units allows a sort of ‘hoof capsule flow’ also known as ‘thermal growth rate’ [[10](#_ENREF_10)] or possibly both.

The hoof capsule, like the skin, is a form of stratified-cornified epithelium [[11](#_ENREF_11)]. In other words, both are formed from layers of epidermal cells, predominantly keratinocytes [[12](#_ENREF_12)], that exist in their basal, proliferative state closest to the BM, where they are nourished by the adjacent highly vascularised dermis, and differentiate sequentially as they travel distally/outward, becoming increasingly distinct from their original state [[4](#_ENREF_4), [13](#_ENREF_13)]. The epidermis of the skin, a stratified soft-cornifying epithelium, consists of 4-5 layers: the *stratum basale*, in which cells are highly proliferative and continually add to the epidermis; the *stratum spinosum*, which consists of column-shaped cells that have commenced the differentiation process; the *stratum granulosum*, in which cells contain keratohyalin granules and lamellar bodies which play roles in the final stages of differentiation, barrier formation, and desquamation; the *statum lucidum*, which exists only in the thickened skin of the palms and soles of the hands and feet and contains eleidin, a product of keratohyalin; and the *stratum corneum*, in which the anucleate cells are terminally differentiated and form a strong, impermeable structure to protect the layers beneath [[13](#_ENREF_13), [14](#_ENREF_14)]. While present in the softer periople and bulbs of the heel, the hoof capsule, along with other stratified hard-cornifying epithelia such as the nail plate and hair cortex, lacks a *stratum granulosum* [[15](#_ENREF_15), [16](#_ENREF_16)]. The *stratum corneum* of stratified soft-cornifying epithelia desquamates continuously, while the hoof is, in the wild, worn down by the constant friction of movement [[11](#_ENREF_11)].

The cells of each epidermal layer express markers specific to their degree of differentiation. Prominent markers in keratinocytes are the keratins (K), of which at least 30 have been identified in horse hoof tissue [[16](#_ENREF_16)]. Keratins are components of the intermediate filament (IF), which provides structural integrity to the cell as well as playing roles in cell signalling and intracellular vesicle transport. The physical properties of a particular keratin protein is given by its molecular weight and its isoelectric point (pI) and giving rise to two classes of keratin – type I, or acidic keratins, and type II, or basic keratins – which heterodimerise during their formation of the IF [[11](#_ENREF_11)]. The most abundantly expressed keratins in the hoof are K124 and its partner K42, both of which are hoof-specific. However, while they are specific makers of hoof keratinocytes, they are expressed in both the basal and suprabasal layers, and thus do not serve as markers of differentiation [[16](#_ENREF_16)]. Furthermore, K124 expression may be limited to the lamellae and may not be expressed in the coronet [[17](#_ENREF_17)]. The Acidic K14 and its basic counterpart K5 are ubiquitously expressed in basal keratinocytes, and are downregulated as the cell differentiates [[12](#_ENREF_12), [16-18](#_ENREF_16)]. K10 and K1 are expressed in the *stratum spinosum* of the skin [[12](#_ENREF_12), [19](#_ENREF_19)]. K10 expression was reported in the suprabasal layers of the cow’s claw [[20](#_ENREF_20)] and K81 is weakly expressed in the hoof [[16](#_ENREF_16)] and has been linked with the advanced stages of differentiation in the hair cortex [[11](#_ENREF_11)].

Components of hemidesmosomes have been localised in the basal layers of the SEL. These include: integrins α6 and β4, which heterodimerise to form a laminin receptor as well as interacting with hemidesmosome plaque proteins; bullous pempigoid antigen 1 (BP180, also known as collagen 17A1 which is found in human skin [[12](#_ENREF_12)]) and laminin-332 (Ln-332, formerly known as laminin-5) which are anchoring filaments; and bullous pempigoid antigen 2 (BP230) and plectin, which are intracellular hemidesmosome plaque proteins [[21](#_ENREF_21), [22](#_ENREF_22)]. Adherens junction components β-catenin and E-cadherin have also been found to be expressed [[21](#_ENREF_21)]. Marking cells with high growth potential (i.e. epidermal stem cells – ESCs – and transit amplifying cells) in the basal epidermal cells of both the skin and the hoof is transcription factor p63, which has been often found co-localised with Ki-67, a marker of active proliferation [[12](#_ENREF_12), [23](#_ENREF_23), [24](#_ENREF_24)].

**
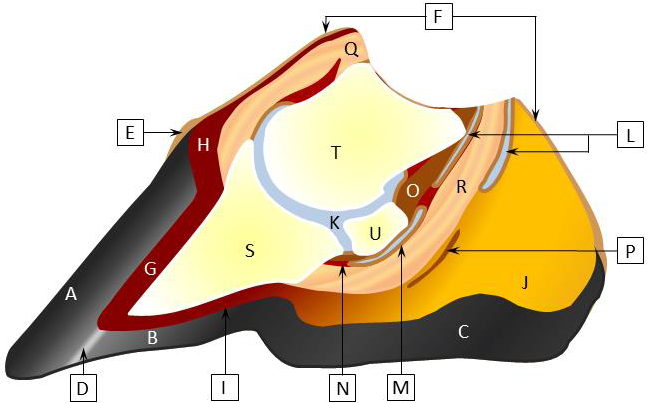
**

**Fig.SM0: Basic hoof anatomy: (A)** Hoof Wall; **(B)** Sole; **(C)** Frog; **(D)**White Line; **(E)** Periople; **(F)** Skin; **(G)** Lamellar Corium; **(H)** Coronary Corium; **(I)** Solar Corium; **(J)** Digital Cushion; **(K)** Distal Interphalangeal Joint; **(L)** Distal Synovial Sheaths; **(M)** Navicular Synovial Bursa; **(N)** Distal Navicular Ligament; **(O)** Navicular Suspensory Ligament; **(P)** Distal Digital Annular Ligament; **(Q)** Common Digital Extensor Tendon; **(R)** Deep Digital Flexor Tendon; **(S)** Third Phalanx; **(T)** Second Phalanx; **(U)** Navicular Bone.

**SM.1: Dorsal hoof curvature measurements**

To measure the dorsal curvature of the hoof two approaches were used depending as to whether the hoof was obtained as abattoir remnant or the horse was alive. Each time the hoof was collected from an abattoir it was scanned using the µCT to obtain its 3D reconstruction (examples of such reconstruction can be found either in <https://www.sv-hoof-project.com/previous-research> for the hoof or <https://www.nottingham.ac.uk/research/groups/physical-mathematical-veterinary-medicine/index.aspx> for the papilla). Using Fiji software it was then possible to re-orientate and slice the hoof in a sagittal way following the dorso-ventral direction until reaching the image positioned exactly at mid-distance between the lateral and medial parts of the hoof. Then the coordinates of the dorsal hoof edge were extracted (see Fig.SM1A). For live horses a lateral picture of their foot was taken as described in 2.6 in the main text, see ‘2. Material and methods’, and using Fiji, a similar determination for the coordinates of the dorsal hoof edge was used (Fig.SM1B). From the list of coordinates $(x,y)$ of the dorsal hoof edge it was then possible to plot the dorsal profile of the hoof and to fit the profile with a polynomial series under the form $y=f(x)$. To determine the local dorsal curvature of the hoof, $C\left( x \right)$, the formula $C\left( x \right)=f''(x)/\left| 1+{f^{'}\left( x \right)}^{\frac{3}{2}} \right|$ was used where |.|, $f^{'}\left( x \right)$ and $f^{''}\left( x \right)$ represent, respectively, the absolute value and, the first and second derivatives of $f\left( x \right)$ with regard to the variable ‘$x$’. Then the average dorsal curvature was determined using the following integral: $\int_{x1}^{x2} C\left( x \right)dx/(x2-x1)$ where $x1 \text{and} x2$ represent the abscises corresponding to the extremities of the dorsal edge of the hoof selected. When the distal edge of the hoof was visibly rasped the distal extremity of the hoof was not included in the analysis.

**Fig.SM1: Dorsal hoof curvature analysis: (A)** the hoof selected from the abattoir were scanned and re-oriented to extract the image at mid-distance between the lateral and medial parts of the hoof. Then the coordinates of the dorsal edge of the hoof were extracted for further analysis. **(B)** For live horses a similar imaging technique was used as in (A) but photographic pictures were taken instead.

**SM.2: Normal and tangential stress components applied on the papillae related to the changes in cell size in the interpapillary space: Synchronization of the transition.**

Stating that cells from the papillae emerge in the interpapillary space only proximally leads to an apparent paradox as Fig.2D (main text) shows that the cells lining the papillae (that have not entered the interpapillary space) are in a more differentiated state when located distally, compared to the same cells located proximally. The ratio between the expressions of K14 and K10 confirms this observation as K14/K10~1 and K14/K10~0.5 can be estimated in the middle and proximal regions, respectively. This suggests that as the cells move from the distal to the proximal regions of the papillae, the expression of K14 is stimulated. Furthermore, if one considers that the sequential expression of K14 and then K10 is indicative of keratinocyte differentiation, these observations suggest that the cells lining the papillae de-differentiate prior to entering the interpapillary space. It would not be a surprise that such a process exists to synchronize the state of cells prior to them entering the interpapillary space, particularly if the soft-to-hard transition occurs as an abrupt transition. This process can potentially be linked to the interpapillary pressure and the shape of the papillae. The interpapillary pressure has a tangential component along the papillary membrane that, given the shape of the papillae, is maximal proximally and vanishes along the papillae (Fig.SM2A). If true, given the symmetry of the papillae, this means that the cells forming the proximal part of papillae are stretched. As the progenitor cell marker K14 is mostly expressed proximally (Fig.2D, main text), the local proximal stretch might be a synchronizing point of hoof morphogenesis.

To assess whether a stretch would stimulate early keratogenesis response(s), a primary culture of horse keratinocyte progenitor cells had to be established. Each time cells were needed, a horse hoof was dissected, as continuous culture was not feasible in order to avoid the use of growth factors that could interfere with the differentiation process. Isolated cells expressed early stage makers of keratogenesis, K14 and CD44 (Fig.SM2B), and demonstrated an increase in their size/surface area, a sign of keratinocyte differentiation, that was visible from the first week onward (Fig.SM2C). Concomitantly, the ratio ‘fluorescence intensity to cell’s surface area’ demonstrated that membrane (CD44) or cytosolic (Ki67, K14) markers of the early stages of keratogenesis dropped after about 2 weeks (Fig.SM2C), one passage is equivalent to 7 to 8 days. However this was not caused by the dilution of the marker due to the increase in cell surface area, as the total fluorescence intensity per cell decreased similarly. These data suggest that any work on keratinocyte progenitor cells has to be performed within two weeks of cell isolation, as otherwise cells start to differentiate. Finally, the pluripotency of isolated cells was also tested using an osteogenic medium. After 19 days incubation in osteogenic medium, most cells (~90%) were osteoblasts, in turn confirming an adequate protocol for the isolation of pluripotent keratinocyte progenitor cells from the papillae (Fig.SM2D).

To measure how a 2D strain can stimulate the expression of keratinocyte markers, a well with a flexible silicon base was used to culture and stretch cells by ~30% over 5 days by placing a marble of chosen radius underneath the well and placing a weight on the top of the well (Fig.SM2E left). To confirm the impact of an isotropic two-dimensional stretch, only cells located at the centre of the well (blue part of the silicon membrane, Fig.SM2E right), were studied. The relationship between the parameters describing the well and the marble and the strain applied can be found in [[25](#_ENREF_25)]. The particular choice for the value of the strain stems from the contraction ~30-50% in the papillae length when dissociated from the hard hoof epidermis (data not shown). Upon stretch it was found that the expression of K14 increases and is maintained over time (Fig.SM2F). To assess whether this was unique to K14 a pan keratin antibody was also used that detects all keratins in the equine species. It was found that the stretch has a notable tendency to decrease keratin expressions levels (on this blot the second band from the top that is stimulated by the stretch corresponds to K14 molecular weight). However the ~37kDa keratin protein seemed to be more affected by stretch (based on the molecular weight the protein is likely K19). The results suggest that stretching cells results in a visible tendency to drop the expression of mature keratin proteins to promote the expression of early progeny keratins and that this event could synchronize cells to fine tune hoof morphogenesis. While the stretch had a tendency to dedifferentiate cells, no Ki-67 nuclear translocation were observed (data not shown).

**Fig.SM2: Impact of papillae shape on cell differentiation. (A)** Sketch showing the tangential and normal stresses linked to the interpapillary pressure. Let us consider a function $F\left(y\right)$ that describes the 2D shape of the papillae along the y-axis and the pressure $P\left(y\right)$ resulting from the cells changing volume. Together $F\left(y\right)$ and $P\left(y\right)$ allow us to determine the component of the interpapillary pressure normal to the papillae, $\sigma_{n}\sim P\left(y\right)/\sqrt{1+\left(dF/dy\right)^{2}}$, that opposes cell entry into the interpapillary space. Similarly, the stress tangent to the papillae can be written as, $\sigma_{t}\sim P\left(y\right)\left(dF/dy\right)/\sqrt{1+\left(dF/dy\right)^{2}}$. The ratio between the two components is $\sigma_{t}/\sigma_{n}\sim\left(dF/dy\right)$. Given the shape of the papillae $\sigma_{t}/\sigma_{n}>>1$ proximally and $\sigma_{t}/\sigma_{n}<<1$ distally that is to say that the normal stress against the papillae is expected to be predominant distally and the tension against the papillae is expected to be predominant proximally. **(B, C)** Visualisation (B) and measure (C) of markers linked to keratinocyte progeny stages. (‘*’: p-value <5% against P1 values). **(D)** Osteogenesis of isolated cells demonstrating their pluripotency. Percentage of positively labelled (red) cells. (‘*’: p-value <5% against control values; standard errors are represented). **(E)** Sketch of the system used to stretch cells. **(F)** Immunofluorescence of K14 when cells are stretched (‘S’) compared to control (‘C’) over a 5 days period. Western blots for K14 and pan-K when cells are stretched (‘S’) compared to control (‘C’) over a 5 days period. (‘*’: p-value <5% against the control value for a given time point; standard errors are represented). All analysis were compared against the house keeping protein beta actin. (‘*’: p-value <5%).

**SM.3: Estimation of $\left(P_{\theta=0}\right)_{0}$ using histology pictures**

As cell division is a rather slow process with an order of magnitude $\tau_{0}\sim10h$, one can assume the thermodynamic equilibrium between the basement membrane of papillae and the pressure, $\left(P_{\theta=0}\right)_{0}$ , in the proximal interpapillary space. Considering the papilla as an incompressible 2D fluid with constant dimensions, any duplicating cells on the papilla will increase the surface energy of the papillae. Let us consider the cylindrical symmetry of the papillae and note by ‘$y$’ the azimuthal axis of the papillae passing through its center (that is similar to the axis used to describe the hoof growth). In this context the total length of the papillae shall be noted, $L$, and the diameter of its cross section, $R_{papi}\left(y\right)$. By noting the rate of cellular proliferation, the variable represents the number of cells proliferating at the ordinate ‘$y$’. Isolating a cross section of the papillae of surface area $S_{papi}\left(y\right)=2\pi R_{papi}\left(y\right)\times2R_{0}$, the duplicating cells in this cross section increase the surface energy locally by a factor, $\sigma_{papi}\left(y\right)=hK\left(\Delta S_{papi}\left(y\right)/S_{papi}\left(y\right)\right)$, where $K$ is the elastic modulus of cells and $h$ the thickness of papillae. Assuming that the proliferation is moderate to warrant a linear approximation, the surface tension can be rewritten as . Thus the resulting total energy associated with the cellular proliferation anywhere on the papillae is therefore: ; where $u=y/2R_{0}$ and$u_{T}=L/2R_{0}$.

Now, let us assume that within the proximal interpapillary space the pressure is $\left(P_{\theta=0}\right)_{0}$. Given the shape of the papillae (Fig.SM2), when cells leave the papillae they can only enter the interpapillary space at the location $u=1$ and the energy increment resulting from their entry is  where $\pi R_{0}^{2}h$ is the volume of a single cell. Equating both energies one finds that the proximal interpapillary pressure must verify:

[1]

From Eq.1 we note that if more cells proliferate on the papillae but that the profile of proliferation is the same, namely that the ratio is constant, then the resulting pressure is expected to remain constant. This is an assumption that we shall consider valid throughout the manuscript. To estimate Eq.1 the data concerning the proliferation of cells on the papillae, Fig.2A, was reused. The y-axis was normalised by the nominal cell size $2R_{0}\sim10\mu m$ and the number of proliferating cells were categorized over 50-cells intervals and normalized by the initial value found (i.e. from the first 50-cells interval). Then the integral was estimated using Newton’s method (Fig.S3). Assuming $K\sim10^{3}Pa$ allows one to estimate $\left(P_{\theta=0}\right)_{0}\sim1.9\times10^{5}Pa\pm4.9\times10^{4}Pa$.

**Fig.SM3: Native growth stress from the papillae:** Normalized number of proliferating cells as a function of the distance along the papillae expressed in 50-cells units.

**SM.4: Variation in the position of the transition as a function of an excess of cells in the interpapillary space.**

By assuming that the cellular differentiation time is constant, the shift in the position of the transition needs to verify: $\int_{1}^{\left(\bar{y}_{\theta}\right)_{c}} 1/\left(\bar{v}_{\theta}\right)_{\left(\left(\bar{N}_{\theta}\right)_{0},\bar{y}_{\theta}\right)}d\bar{y}_{\theta}\sim\int_{1}^{\left(\bar{y}_{\theta=0}\right)_{c}} 1/\left(\bar{v}_{\theta}\right)_{\left(1,\bar{y}_{\theta}\right)}d\bar{y}_{\theta}$; where.

By assuming small variations and making use of the chain rule method the growth rate can be rewritten as: $\left(1/\bar{v}_{\theta}\right)_{\left(\left(\bar{N}_{\theta}\right)_{0},\bar{y}_{\theta}\right)}\sim\left(1/\bar{v}_{\theta}\right)_{\left(1,\bar{y}_{\theta}\right)}+\left(\partial\left(1/\bar{v}_{\theta}\right)/\partial\bar{N}_{\theta}\right)_{\left(1,\bar{y}_{\theta}\right)}\left[\left(\bar{N}_{\theta}\right)_{0}-1\right]$. Therefore by noting $\bar{v}_{\theta}=v_{\theta}/v_{0}$ the integrals can be simplified and rewritten as,

$\left(\bar{y}_{\theta}\right)_{c}-\left(\bar{y}_{\theta=0}\right)_{c}\sim\left[\left(\bar{N}_{\theta}\right)_{0}-1\right]\left(\bar{v}_{\theta}\right)_{c}\int_{1}^{\left(\bar{y}_{\theta=0}\right)_{c}} \left(\partial\left(1/\bar{v}_{\theta}\right)/\partial\bar{N}_{\theta}\right)_{\left(1,\bar{y}_{\theta}\right)}d\bar{y}_{\theta}$.

Finally it follows:

$\begin{matrix} \left(\bar{y}_{\theta}\right)_{c}/\left(\bar{y}_{\theta=0}\right)_{c}-1\sim\chi\times\left[\left(\bar{N}_{\theta}\right)_{0}-1\right] \\ \chi=-\frac{\lambda v_{0}}{3K}\left(\bar{v}_{\theta}\right)_{c}\frac{\left(\left(\bar{y}_{\theta=0}\right)_{c}-1\right)^{2}}{\left(\bar{y}_{\theta=0}\right)_{c}}\int_{0}^{1} u\left(1+\frac{\lambda v_{0}}{K}\left(\left(\bar{y}_{\theta=0}\right)_{c}-1\right)u\right)^{-\frac{2}{3}}\left(1+4\frac{v_{m}}{v_{0}}\left(\left(\bar{y}_{\theta=0}\right)_{c}-1\right)u\right)^{-\frac{1}{2}}du \end{matrix}$ [2]

In Eq.2 the integral can be evaluated with a numerical value ~0.02 leading to$\chi\sim-0.62$.

**SM.5 Variations in the dorsal growth rate of the hoof as a function of chronic changes in the animal’s weight or excessive loading.**

To estimate the impact of the animal’s weight on the growth rate of its hooves, let us consider straight hooves ($C=0$) and concentrate on the dorsal region, i.e. $\theta=0$. Two versions of Eq.3 from the main text can then be used, the first one when the horse weight imposes a nominal load $\rho_{0}$ to which correspond a growth rate $\left(v_{\theta=0}\right)_{c}^{0}$ and the second one when the load has changed from $\rho_{0}$ to $\rho$ leading to a change in the growth rate from $\left(v_{\theta=0}\right)_{c}^{0}$ to $\left(v_{\theta=0}\right)_{c}$. Prior to writing the balances of stresses it is essential to determine how the change in growth rate is linked to an excess of interpapillary cells. Indeed, the change in the growth rate is linked to a change in the amount of cells inside the interpapillary space from $\left(\bar{N}_{\theta=0}\right)_{0}=1$ to $\bar{N}_{\theta=0}$, resulting also in a change in the position of the transition from $\left(\bar{y}_{\theta=0}\right)_{c}^{0}$ to $\left(\bar{y}_{\theta=0}\right)_{c}$. In the context of small variations the growth rate $\left(v_{\theta=0}\right)_{c}$ can be approximated by $\left(v_{\theta=0}\right)_{c}/\left(v_{\theta=0}\right)_{c}^{0}\sim1+\left(\alpha+\beta\chi\right)\left(\bar{N}_{\theta=0}-1\right)$ where $\chi\sim-0.62$ has been defined in SM.4, $\alpha=\left(1/\left(v_{\theta=0}\right)_{c}^{0}\right)\times\left.\left[\partial_{\bar{N}_{\theta=0}}\left(v_{\theta=0}\right)_{c}\right]\right|_{\left(\left(\bar{y}_{\theta=0}\right)_{c}=\left(\bar{y}_{\theta=0}\right)_{c}^{0}.\bar{N}_{\theta=0}=1\right)}$and $\beta=\left(\left(\bar{y}_{\theta=0}\right)_{c}^{0}/\left(v_{\theta=0}\right)_{c}^{0}\right)\times\left.\left[\partial_{\left(\bar{y}_{\theta=0}\right)_{c}}\left(v_{\theta=0}\right)_{c}\right]\right|_{\left(\left(\bar{y}_{\theta=0}\right)_{c}=\left(\bar{y}_{\theta=0}\right)_{c}^{0}.\bar{N}_{\theta=0}=1\right)}$. Both derivatives can be determined numerically using Eq.1 (main text) leading to:

$$\begin{matrix} \alpha=\frac{\lambda v_{0}}{3K}\frac{\left(\bar{y}_{\theta=0}\right)_{c}^{0}-1}{1+\frac{\lambda v_{0}}{K}\left(\left(\bar{y}_{\theta=0}\right)_{c}^{0}-1\right)}\sim0.31 \\ \beta=2\frac{v_{m}}{v_{0}}\frac{\left(\bar{y}_{\theta=0}\right)_{c}^{0}}{1+4\frac{v_{m}}{v_{0}}\left(\left(\bar{y}_{\theta=0}\right)_{c}^{0}-1\right)}-\frac{\lambda v_{0}}{3K}\frac{\left(\bar{y}_{\theta=0}\right)_{c}^{0}}{1+\frac{\lambda v_{0}}{K}\left(\left(\bar{y}_{\theta=0}\right)_{c}^{0}-1\right)}\sim0.19 \end{matrix}$$

Thus the two balances of stresses aforementioned can be rewritten:

$e\left[\left(P_{\theta=0}\right)_{0}-\lambda v_{0}\left(\left(\bar{y}_{\theta=0}\right)_{c}^{0}-1\right)\right]=f_{adh}^{0}\left(v_{\theta=0}\right)_{c}^{0}Z_{\theta=0}+\rho_{0}ge\Omega\left(0\right)$ [3]

$e\left[\left(P_{\theta=0}\right)_{0}-\lambda v_{0}\left(\left(\bar{y}_{\theta=0}\right)_{c}-1\right)/\bar{N}_{\theta=0}\right]=f_{adh}^{0}\left(v_{\theta=0}\right)_{c}Z_{\theta=0}+\rho ge\Omega\left(0\right)$ [4]

Subtracting the balances of stresses by using first order approximations allows one to write:

$\left[1-\frac{e\lambda v_{0}\left(\bar{y}_{\theta=0}\right)_{c}^{0}\left(1-\chi\right)}{Z_{\theta=0}f_{adh}^{0}\left(v_{\theta=0}\right)_{c}^{0}\left(\alpha+\beta\chi\right)}\right]\frac{\delta\left(\bar{v}_{\theta=0}\right)_{c}}{\left(\bar{v}_{\theta=0}\right)_{c}^{0}}\sim-\frac{\delta\rho}{\rho_{0}}\frac{\rho_{0}ge\Omega\left(0\right)}{Z_{\theta=0}f_{adh}^{0}\left(v_{\theta=0}\right)_{c}^{0}}$ [5]

The *lhs* term within bracket can be estimated ~0.65 and is therefore positive that implies that the optimal relation for a straight hoof, i.e. $\delta\left(v_{\theta=0}\right)_{c}\sim+\delta\rho$, is not fulfilled and changes in BCS should result in a curved hoof over time.

**SM.6 Variation in the growth rate of the hoof and the dorsal curvature of the hoof as a function of the loading of the animal.**

As the growth stress seems to be homogenous across the coronet (Fig. 3B, main text) and that the quarter regions are shorter than the dorsal part of the hoof, blocking hoof growth at the dorsal region does not mean blocking hoof growth at the quarters, as the adhesion stress is lower in the quarter regions. Thus dished hooves are expected to reappear beyond this threshold loading. To determine this critical loading, let us assume a working equids with straight hoof initially and consider now that the working Equids is loaded excessively and that the hoof compensates for this loading. The relations obtained in SM.5 are therefore valid. In this context it is possible to define a non-dimensioned parameter, $\left(\delta\rho/\rho_{0}\right)_{c}$, representing the load at which point the dorsal growth of the hoof stops, which by using $\Omega\left(0\right)\sim\cos\left(40^{o}\right)$ [[26](#_ENREF_26)] can be numerically estimated with the lowest order approximations used so far:

$\left(\frac{\delta\rho}{\rho_{0}}\right)_{c}=\left[1-\frac{e\lambda v_{0}\left(\bar{y}_{\theta=0}\right)_{c}^{0}\left(1-\chi\right)}{Z_{\theta=0}f_{adh}^{0}\left(v_{\theta=0}\right)_{c}^{0}\left(\alpha+\beta\chi\right)}\right]\times\left[\frac{\rho_{0}ge\Omega\left(0\right)}{Z_{\theta=0}f_{adh}^{0}\left(v_{\theta=0}\right)_{c}^{0}}\right]^{-1}\sim1.3$ [6]

Thus loading a working equids ~1.3 its own body weight should result in dished hooves. Using $\rho_{0}\sim1.6\times10^{4}kg/m^{2}$ allows one to obtain a critical loading value $\sim3.7\times10^{4}kg/m^{2}$. Table SM1 provides the weight range across the equids species and the load mass. Remarkably, smaller equids carry more load than larger ones.

**Table SM1: Typical weight of working equids, working hours per day and the weight of the load the working equids must carry.**

| **Species** | **Mass range (kg)** | **Working hours per day** | **Load mass (kg)** | **Ref** |
| --- | --- | --- | --- | --- |
| **Horse** | 380-1000 | 8 | 137-415 | [[27-29](#_ENREF_27)] |
| **Donkey** | 80-480 | 6-12 | 137-1000 | [[27](#_ENREF_27), [30-33](#_ENREF_30)] |
| **Mule** | 23-450 | 8-12 | 137-1200 | [[32](#_ENREF_32), [34](#_ENREF_34), [35](#_ENREF_35)] |

**SM.7: Ki-67 nuclear localisation upon insulin stimulation of keratinocyte progenitor cells**

To effectively measure the impact of insulin on Ki-67, at P1 keratinocytes were trypsinized and seeded into 12-well culture plates at a density of 2×10^5^ cells/well for 24hr. Cells were then starved for 24hrs in DMEM-F-12 containing 1% FCS only prior to being with insulin (Insulin solution from bovine pancreas, Sigma, USA) at a concentration of 1µg/ml or 100ng/ml for 2, 8, 24 and 48hrs before fixation (4% PFA/PBS). Control cells were not incubated with insulin over the experimental times (Fig.SM4).

**Fig.SM4: Insulin stimulation of progenitor keratinocyte cells.** Ki-67 expression levels and nuclear localisation upon stimulation with insulin. Left: double labelling of Ki-67 and K14 at 24 hrs following insulin stimulation. Right: Measurements of the nuclear intensity of Ki-67 over time following insulin stimulations (‘*’: p-value <5% against control values; standard errors are represented).

**SM.9: Expected impact of high blood insulin level on the hoof dorsal curvature**

Let us consider an Equids of constant mass that, through a biological stimulation like insulin, has a homogenous increase in the amount of interpapillary cells along the coronet. The question that is therefore asked is how such an increase can affect the growth rate of the hoof whatever the angular position considered. We assume that the hoof is initially straight namely that the initial angular growth rate is, $\left(v_{\theta}\right)_{c}^{0}=\left(v_{\theta=0}\right)_{c}^{0}$, and that the endogenous initial profile of cells inside the interpapillary space for any particular angular position is defined by $\left(\bar{N}_{\theta}\right)_{0}=\left(N_{\theta}\right)_{0}/\left(N_{\theta=0}\right)_{0}$. In this context the balance of stresses for any angular position is also:

$e\left[\left(P_{\theta}\right)_{0}-\lambda v_{0}\left(\left(\bar{y}_{\theta}\right)_{c}^{0}-1\right)\right]=f_{adh}^{0}Z_{\theta=0}\gamma_{\theta}\left(v_{\theta}\right)_{c}^{0}+\rho ge\Omega\left(0\right)$ [7]

Now, if an excess $\Delta N$ of cells is injected by the papillae into the interpapillary space at the same angular position, the growth stress and the growth rate are altered and the new balance of stresses becomes:

$e\left[\left(P_{\theta}\right)_{0}-\frac{\lambda v_{0}\left(\left(\bar{y}_{\theta}\right)_{c}-1\right)}{\frac{\left(N_{\theta}\right)_{0}+\Delta N}{\left(N_{\theta}\right)_{0}}}\right]=f_{adh}^{0}Z_{\theta=0}\gamma_{\theta}\left(v_{\theta}\right)_{c}+\rho ge\Omega\left(0\right)$ [8]

If one further assumes that $\left(\bar{y}_{\theta}\right)_{c}>>1$, $\Delta N/\left(N_{\theta}\right)_{0}<<1$ and that $\left(P_{\theta}\right)_{0}$ does not change (SM.3, SM Appendix), by subtracting Eq.7 from Eq.8 one finds at the leading order in $\Delta N$:

$-e\lambda v_{0}\left[\left(\bar{y}_{\theta}\right)_{c}-\left(\bar{y}_{\theta}\right)_{c}^{0}\right]+e\lambda v_{0}\left(\bar{y}_{\theta}\right)_{c}\left(\Delta N/\left(N_{\theta}\right)_{0}\right)\sim f_{adh}^{0}\left(v_{\theta}\right)_{c}^{0}Z_{\theta=0}\gamma_{\theta}\left(\delta\left(v_{\theta}\right)_{c}/\left(v_{\theta}\right)_{c}^{0}\right)$ [9]

Where $\delta\left(v_{\theta}\right)_{c}=\left(v_{\theta}\right)_{c}-\left(v_{\theta}\right)_{c}^{0}$. Making use of SM.5 (SM Appendix) for a given angular position that is, $\left(\bar{y}_{\theta}\right)_{c}-\left(\bar{y}_{\theta}\right)_{c}^{0}\sim\left(\bar{y}_{\theta}\right)_{c}^{0}\chi\left(\Delta N/\left(\bar{N}_{\theta}\right)_{0}\right)$; and incorporating the latter relation into Eq.9 leads to:

$e\lambda v_{0}\left(\bar{y}_{\theta}\right)_{c}^{0}\left(1-\chi\right)\frac{\Delta N}{\left(N_{\theta}\right)_{0}}\sim f_{adh}^{0}\left(v_{\theta}\right)_{c}^{0}Z_{\theta=0}\gamma_{\theta}\frac{\delta\left(v_{\theta}\right)_{c}}{\left(v_{\theta}\right)_{c}^{0}}$ [10]

In Eq.10 one notes that as $\left(v_{\theta}\right)_{c}^{0}=\left(v_{\theta=0}\right)_{c}^{0}$, it is useful to introduce the physical variables related to the origin of the angular position, namely $\left(\bar{y}_{\theta=0}\right)_{c}^{0}$ and $\left(N_{\theta=0}\right)_{0}$, so that the local growth rate whatever angular position can be compared to the dorsal growth rate. Eq.10 can then be rewritten:

$e\lambda v_{0}\left(\bar{y}_{\theta=0}\right)_{c}^{0}\frac{\left(\bar{y}_{\theta}\right)_{c}^{0}}{\left(\bar{y}_{\theta=0}\right)_{c}^{0}}\left(1-\chi\right)\frac{1}{\left(\bar{N}_{\theta}\right)_{0}}\frac{\Delta N}{\left(N_{\theta=0}\right)_{0}}\sim f_{adh}^{0}\left(v_{\theta=0}\right)_{c}^{0}Z_{\theta=0}\gamma_{\theta}\frac{\delta\left(v_{\theta}\right)_{c}}{\left(v_{\theta=0}\right)_{c}^{0}}$ [11]

Using Eq.3 (SM.3, SM Appendix) and the literal expression of $\left(\bar{N}_{\theta}\right)_{0}$ (see main text), the leading order in $\Delta N$ becomes:

$\begin{matrix} \left[\frac{1+\omega}{\omega\gamma_{\theta}}-1\right]\left(1-\chi\right)^{2}\frac{\Delta N}{\left(N_{\theta=0}\right)_{0}}\sim\frac{\delta\left(v_{\theta}\right)_{c}}{\left(v_{\theta=0}\right)_{c}^{0}} \\ \omega=\left(1-\chi\right)\frac{f_{adh}^{0}\left(v_{\theta=0}\right)_{c}^{0}Z_{\theta=0}}{e\lambda v_{0}\left(\bar{y}_{\theta=0}\right)_{c}^{0}}\sim40 \end{matrix}$ [12]

As a result, when more cells enter the interpapillary space homogeneously across the coronary band the growth rate diverges as a function of the angular position considered.

**Supplementary Bibliography**

[1] H. Bragulla, Fetal development of the segment-specific papillary body in the equine hoof, Journal of morphology, 258 (2003) 207-224.

[2] C.C. Pollitt, The anatomy and physiology of the hoof wall, Equine Vet Educ, 10 (1998) 318-325.

[3] C.C. Pollitt, The anatomy and physiology of the suspensory apparatus of the distal phalanx, The Veterinary clinics of North America. Equine practice, 26 (2010) 29-49.

[4] M. Daradka, C.C. Pollitt, Epidermal cell proliferation in the equine hoof wall, Equine Vet J, 36 (2004) 236-241.

[5] D. Rayneau-Kirkhope, Y. Mao, C. Rauch, Bioinspired Hierarchical Designs for Stiff, Strong Interfaces between Materials of Differing Stiffness, Phys Rev Appl, 10 (2018).

[6] C.C. Pollitt, Anatomy and physiology of the inner hoof wall, Clinical Techniques in Equine Practice, 3 (2004) 3-21.

[7] J.E. Stump, Anatomy of the normal equine foot, including microscopic features of the laminar region, J Am Vet Med Assoc, 151 (1967) 1588-1598.

[8] C. Rauch, M. Cherkaoui-Rbati, Physics of nail conditions: why do ingrown nails always happen in the big toes?, Phys Biol, 11 (2014).

[9] S. Pal-Ghosh, T. Blanco, G. Tadvalkar, A. Pajoohesh-Ganji, A. Parthasarathy, J.D. Zieske, M.A. Stepp, MMP9 cleavage of the beta 4 integrin ectodomain leads to recurrent epithelial erosions in mice, Journal of Cell Science, 124 (2011) 2666-2675.

[10] C. Rauch, M. Cherkaoui-Rbati, Physics of nail conditions: why do ingrown nails always happen in the big toes?, Physical biology, 11 (2014) 066004.

[11] H.H. Bragulla, D.G. Homberger, Structure and functions of keratin proteins in simple, stratified, keratinized and cornified epithelia, J Anat, 214 (2009) 516-559.

[12] P.H. Edqvist, L. Fagerberg, B.M. Hallstrom, A. Danielsson, K. Edlund, M. Uhlen, F. Ponten, Expression of human skin-specific genes defined by transcriptomics and antibody-based profiling, J Histochem Cytochem, 63 (2015) 129-141.

[13] D.D. Bikle, Z. Xie, C.L. Tu, Calcium regulation of keratinocyte differentiation, Expert Rev Endocrinol Metab, 7 (2012) 461-472.

[14] H. Yousef, S. Sharma, Anatomy, Skin (Integument), Epidermis, in: StatPearls, Treasure Island (FL), 2018.

[15] H.H. Bragulla, D.G. Homberger, The role of the specific, profilaggrin-containing keratohyalin granules in the developing epidermis of the fetal horse hoof, Pferdeheilkunde, 23 (2007) 5-+.

[16] R.A. Carter, V. Shekk, M.A. de Laat, C.C. Pollitt, H.L. Galantino-Homer, Novel keratins identified by quantitative proteomic analysis as the major cytoskeletal proteins of equine (Equus caballus) hoof lamellar tissue, Journal of animal science, 88 (2010) 3843-3855.

[17] R.L. Linardi, S.O. Megee, S.R. Mainardi, M. Senoo, H.L. Galantino-Homer, Expression and localization of epithelial stem cell and differentiation markers in equine skin, eye and hoof, Vet Dermatol, 26 (2015) 213-e247.

[18] H. Alam, L. Sehgal, S.T. Kundu, S.N. Dalal, M.M. Vaidya, Novel function of keratins 5 and 14 in proliferation and differentiation of stratified epithelial cells, Mol Biol Cell, 22 (2011) 4068-4078.

[19] C. Aguiar, J. Therrien, P. Lemire, M. Segura, L.C. Smith, C.L. Theoret, Differentiation of equine induced pluripotent stem cells into a keratinocyte lineage, Equine Vet J, 48 (2016) 338-345.

[20] K.A. Hendry, A.J. MacCallum, C.H. Knight, C.J. Wilde, Synthesis and distribution of cytokeratins in healthy and ulcerated bovine claw epidermis, J Dairy Res, 68 (2001) 525-537.

[21] L. Wang, E.A. Pawlak, P.J. Johnson, J.K. Belknap, S. Eades, S. Stack, H. Cousin, S.J. Black, Impact of laminitis on the canonical Wnt signaling pathway in basal epithelial cells of the equine digital laminae, PLoS One, 8 (2013) e56025.

[22] K.R. French, C.C. Pollitt, Equine laminitis: cleavage of laminin 5 associated with basement membrane dysadhesion, Equine Vet J, 36 (2004) 242-247.

[23] H. Green, K. Easley, S. Iuchi, Marker succession during the development of keratinocytes from cultured human embryonic stem cells, Proc Natl Acad Sci U S A, 100 (2003) 15625-15630.

[24] R.A. Carter, J.B. Engiles, S.O. Megee, M. Senoo, H.L. Galantino-Homer, Decreased expression of p63, a regulator of epidermal stem cells, in the chronic laminitic equine hoof, Equine Vet J, 43 (2011) 543-551.

[25] C. Rauch, P.T. Loughna, Static stretch promotes MEF2A nuclear translocation and expression of neonatal myosin heavy chain in C2C12 myocytes in a calcineurin- and p38-dependent manner, American journal of physiology. Cell physiology, 288 (2005) C593-605.

[26] S.J. Dyson, C.A. Tranquille, S.N. Collins, T.D.H. Parkin, R.C. Murray, An investigation of the relationships between angles and shapes of the hoof capsule and the distal phalanx, Equine Vet J, 43 (2011) 295-301.

[27] A.S. Aluja, The welfare of working equids in Mexico, Applied Animal Behaviour Science, 59 (1998) 19-29.

[28] T. Tadich, A. Escobar, R.A. Pearson, Husbandry and welfare aspects of urban draught horses in the south of Chile, Archivos de Medicina Veterinaria, 40 (2008) 267-273.

[29] C.E. Broster, C.C. Burn, A.R. Barr, H.R. Whay, The range and prevalence of pathological abnormalities associated with lameness in working horses from developing countries, Equine Vet J, 41 (2009) 474-481.

[30] G. Girma, A. Samuel, K. Pascal Gitari, Performance and Welfare Status of Working Donkeys, Journal of Agricultural Science and Technology A, 6 (2016).

[31] M.M. Diarra, A. Doumbia, A.K. McLean, Survey of working conditions and management of donkeys in Niono and Segou, Mali, Proc. ASAS Annual Mtg., 85 (2007).

[32] A.B.A. Ali, M.A.E. Sayed, A.K. McLean, C.R. Heleski, Aggression in working mules and subsequent aggressive treatment by their handlers in Egyptian brick kilns—Cause or effect?, Journal of Veterinary Behaviour, (2018).

[33] W. J.Swann, Improving the welfare of working equine animals in developing countries, Applied Animal Behaviour Science, 100 (2006) 148-151.

[34] W.H. Welfare, 7th International Colloquium on Working Equids, in: J.F. Wade (Ed.), Norwich, UK, 2014.

[35] A. Ali, S. Orion, T. Tesfaye, J.A. Zambriski, The prevalence of lameness and associated risk factors in cart mules in Bahir Dar, Ethiopia, Tropical Animal Health and Production, 48 (2016) 1483-1489.
